# PDIA5 is Correlated With Immune Infiltration and Predicts Poor Prognosis in Gliomas

**DOI:** 10.3389/fimmu.2021.628966

**Published:** 2021-02-16

**Authors:** Hao Zhang, Jialin He, Ziyu Dai, Zeyu Wang, Xisong Liang, Fengqiong He, Zhiwei Xia, Songshan Feng, Hui Cao, Liyang Zhang, Quan Cheng

**Affiliations:** ^1^Department of Neurosurgery, Xiangya Hospital, Central South University, Changsha, China; ^2^Department of Neurology, The Second Xiangya Hospital, Central South University, Changsha, China; ^3^Clinical Diagnosis and Therapy Center for Glioma of Xiangya Hospital, Central South University, Changsha, China; ^4^Department of Neurology, Hunan Aerospace Hospital, Changsha, China; ^5^Department of Psychiatry, The Second People’s Hospital of Hunan Province, The Hospital of Hunan University of Chinese Medicine, Changsha, China; ^6^National Clinical Research Center for Geriatric Disorders, Xiangya Hospital, Central South University, Changsha, China; ^7^Department of Clinical Pharmacology, Xiangya Hospital, Central South University, Changsha, China

**Keywords:** gliomas, PDIA5, immune infiltration, immunotherapy, scRNA-seq

## Abstract

Gliomas are the most common and lethal primary malignant tumor of the brain. Routine treatment including surgical resection, chemotherapy, and radiotherapy produced limited therapeutic effect, while immunotherapy targeting the glioma microenvironment has offered a novel therapeutic option. PDIA5 protein is the member of PDI family, which is highly expressed in glioma and participates in glioma progression. Based on large-scale bioinformatics analysis, we discovered that PDIA5 expression level is upregulated in aggressive gliomas, with high PDIA5 expression predicting poor clinical outcomes. We also observed positive correlation between PDIA5 and immune infiltrating cells, immune related pathways, inflammatory activities, and other immune checkpoint members. Patients with high PDIA5 high-expression benefited from immunotherapies. Additionally, immunohistochemistry revealed that PDIA5 and macrophage biomarker CD68 were upregulated in high-grade gliomas, and patients with low PDIA5 level experienced favorable outcomes among 33 glioma patients. Single cell RNA sequencing exhibited that PDIA5 was in high level presenting in neoplastic cells and macrophages. Cell transfection and co-culture of glioma cells and macrophages revealed that PDIA5 in tumor cells mediated macrophages exhausting. Altogether, our findings indicate that PDIA5 overexpression is associated with immune infiltration in gliomas, and may be a promising therapeutic target for glioma immunotherapy.

## Introduction

Gliomas are the most common primary malignant tumor of the central nervous system in adults and are responsible for most of the deaths caused by primary brain tumors (1, 2), among which glioblastoma multiforme (GBM) is the deadliest subtype. Comprehensive therapy including surgery, radiotherapy, and chemotherapy fails to achieve satisfactory therapeutic effect, and poor survival of GBM patients is associated with the high infiltration of tumor cells and persistence of chemotherapy-resistant cells (3, 4). Recent studies demonstrate that infiltration of immune cells into tumor regions contributes to the development of metastasis and resistance to cancer therapies in gliomas (5, 6). Therefore, to explore other effective treatment options, more work on immunotherapy targeting the glioma microenvironment is being conducted (7–9), and single cell sequencing is providing a new approach to identify immune biomarkers of gliomas (10). Currently, human gliomas are diagnosed using morphological and molecular biomarker criteria according to the 2016 World Health Organization (WHO) classification of central nervous system (CNS) tumors (11). Therefore, further exploration of novel biomarkers to dissect glioma subtypes may help to clarify the molecular mechanisms and promote therapeutic strategies.

Protein disulfide isomerase (PDI), first discovered in 1963, is a 57-kDa dithiol-disulfide oxidoreductase with isomerase and chaperone functions (12, 13). The human PDI gene family currently comprises 21 genes, which have different biochemical characteristics, but share a common structural feature, the TRX-like domain. PDI family proteins are largely expressed in the endoplasmic reticulum (ER) (14, 15), where they play an important regulatory role in protein homeostasis, but also may participate in tumor progression. Previous studies have shown that PDI family protein overexpression correlates with the occurrence, invasion, and metastasis of a variety of malignant tumors (16–20). Consequently, PDI family proteins are likely prognostic factors and therapeutic targets for related tumors (21, 22). Two recent studies have demonstrated that the PDI family may serve as potential prognostic signature in gliomas (16, 23), and PDIA6 (17), P4HB, and PDIA3 (24) have all been proven to be involved in glioma progression. Moreover, high P4HB level contributes significantly to temozolomide resistance (25).

Protein disulfide isomerase A5 (PDIA5), also known as protein disulfide isomerase-related protein (PDIR), is a member of the PDI gene family and also exhibits chaperone-like activity. PDIA5 was first identified in 1995 and was found to be expressed in the brain, liver, kidney, and lungs (26). In gliomas, PDIA5 had significantly increased expression in gliomas compared with normal brain tissues (16).

Currently, the role of PDI proteins in tumor progression mainly lies in their ability to improve tumor apoptosis resistance (19, 27), while other molecular mechanism remains largely unclear. PDIA5 regulates the unfolded protein response (UPR) signaling pathway by activating ATF6α (28), whereby UPR regulates tumor cell survival. Other research has found that PDI inhibition could impair tumorigenic T cells and enhance normal T cell function (29). Based on the aforementioned findings, we speculated that PDIA5 correlated with histopathology grades and immune infiltration of gliomas, and could be a potential prognostic molecule.

In the present study, we comprehensively analyzed the PDIA5 expression pattern in gliomas. We conducted large-scale bioinformatics analyses, using gene expression data downloaded from existing databases, including single cell RNA-sequencing databases. We also performed PDIA5 over-expression and siRNA on U251 then co-culturing with HMC3 *in vitro* to mimic the infiltration of residential immune cells in glioma microenvironment. Additionally, we systematically evaluated the prognostic value of PDIA5 in gliomas. PDIA5 was found to be upregulated in gliomas and related to the suppressive tumor microenvironment by recruiting M2 macrophages, indicating that PDIA5 might be a potential prognostic biomarker or therapy target in the clinical treatment of gliomas.

## Materials and Methods

### Ethics Statement

The experiments were undertaken with the understanding and written consent of each subject. The study methodologies conformed to the standards set by the Declaration of Helsinki, and the study methodologies were approved by the Ethics Committee of Xiangya Hospital, Central South University.

### Clinical Specimens and Data Collection

Archived paraffin embedded glioma tissues (WHO grades I–IV) were collected from patients (n= 31) who underwent surgery in the Department of Neurosurgery, Xiangya Hospital of Central South University. Normal brain tissue samples (n = 3) were gathered from severe traumatic brain injury patients who underwent partial resection of the normal brain.

We obtained data for 1,013 samples from Chinese Glioma Genome Atlas (CGGA) database (http://www.cgga.org.cn/) and 672 samples from The Cancer Genome Atlas (TCGA) database (https://portal.gdc.cancer.gov/). PDIA5 expression data in different radiographical regions of normal brain and GBM were obtained from the Gill dataset (30). RNA-seq data about specific tumor anatomy in GBM was downloaded from the Ivy Glioblastoma Atlas Project (http://glioblastoma.alleninstitute.org/). Single-cell expression matrices were acquired from the Gene Expression Omnibus (GEO; https://www.ncbi.nlm.nih.gov/geo/) GSE138794 (31), and eight single-cell RNA sequencing (scRNA-seq) datasets including both low grade glioma (LGG) and GBM were selected for analysis. Data of immunotherapeutic cohorts was downloaded from IMvigor210 (http://research-pub.Gene.com/IMvigor210CoreBiologies) (32) and GSE78220 in GEO (33).

### Survival Analysis in Kaplan-Meier Plotter

Kaplan-Meier Plotter (https://kmplot.com/analysis/) was used to evaluate the correlation between PDIA5 and survival in across cancer types (34). Briefly, the patient samples were divided into two cohorts according to the cut-off expression of the gene (high *vs.* low expression) for the purpose of assessing prognostic value of PDIA5. We analyzed the relationship of PDIA5 expression with overall survival (OS) and disease specific survival (DSS) in each available cancer type (total number = 33). Hazard ratios (HRs) with 95% confidence intervals (CI) and log-rank P values were calculated.

### Bioinformatics Analysis

The cut-off point was calculated using the R package survminer for OS, progression-free interval (PFI), and DSS. Somatic copy number variations (CNVs) and somatic mutations were downloaded from the TCGA database. CNVs associated with PDIA5 expression were analyzed using GISTIC 2.0 (35). Correlation analysis of PDIA5 was performed for gene expression profiles available in the TCGA and CGGA datasets using the R language. ESTIMATE (Estimation of Stromal and Immune cells in Malignant Tumor tissues using Expression) algorithm was performed as previously reported (36) to evaluate the presence of stromal cells and the infiltration of immune cells in tumor samples. Gene set variation analysis (GSVA) analysis was performed as described in the previous study (37). Briefly, the differential expression in immune cell lineages, GO terms of immune related biological process and inflammatory metagenes from TCGA and CGGA samples were analyzed *via* GSVA. Besides, T cell-inflamed gene expression profile (GEP) levels, cytolytic activity (CYT) was also analyzed through GSVA as described by Ye et al. (38).

### Single-Cell RNA Sequencing

scRNA-seq was performed as described in previous studies (39, 40). The single-cell data expression matrix was processed with the R package Seurat. First, the data was normalized using the “NormalizeData” function, then the function “FindVariableGenes” was used to identify 2,000 highly variable genes. Next, “FindIntergrationAnchors” and “Integratedata” functions were used to merge eight glioma sample datasets. Afterward, the “RunPCA” function was performed and a K-nearest neighbor graph was constructed based on principal component analysis (PCA) using the “FindNeighbors” function, and then the “FindClusters” function was used to alternately combine cells together at the best resolution. Finally, “UMAP” was used for visualization. The “Single R” R package was used to identify the cell types. We chose a glioma dataset in GEO (GSE84465) and data in the Human Primary Cell Atlas Data as a reference. “FeaturePlot” and “VlnPlot” were used to further visualize gene expression. Single-cell pseudotime trajectories reconstruction and analysis was conducted using Monocle according to Pang et al. (41). Briefly, Single cells were projected onto low-dimensional space and ordered into a trajectory with branch points and cells in the same segment of the trajectory were classified as having the same “state”. Additionally, functional annotations by gene set enrichment analysis (GSEA) for PDIA5 in each ‘state’ was constructed. Gene ontology (GO) enrichment analysis and pathway analysis based on Kyoto Encyclopedia of Genes and Genomes (KEGG) was also carried out.

### Immunohistochemistry

Immunohistochemical (IHC) staining was performed as previously described (42). Briefly, sections were obtained from formalin-fixed, paraffin-embedded tissues of normal brains and different grades of human gliomas (WHO grades I–IV). After antigen retrieval and blocking endogenous HRP activity, the slides were blocked with 10% normal goat serum and incubated with primary antibody (anti-PDIA5 antibody human reactivity (D225376, 1:200, Sangon Biotech, China), anti-CD68 E11 human reactivity (SC-17832, 1:400, Santa Cruz, US) at 4°C overnight. Then the signal was visualized using standard protocols. For negative controls, sections were incubated with antibody dilution solution. Slides were counterstained with hematoxylin, and representative images were obtained using an Olympus inverted microscope. H-score of each sample was calculated.

### Cell Transfection and Co-Cultured Organoids

U251 VCT/PDIA5 and U251 siNC/siPDIA5 were co-cultured with HMC3 GFP in 3D condition. In brief, PDIA5 over expression and Vector (VCT) plasmids were transfected *via* Lipofectamine 3000 (Invitrogen, US). Simultaneously. siNC and siPDIA5 RNA transfections were performed *via* RNA Max (Invitrogen, US). Two days post-transfection, tumor, and HMC3 GFP cells were genteelly digested and counted at 5x10’/each, then mixing in 200 µl organoids medium. U251 VCT/PDIA5-HMC3 GFP and U251 siNÇ/siPDIA5-HMC3 GFP in organoids medium were divided and planted 40 ul/droplet. Three days post-plantation, droplets were monitored and imaged by EVOS M5000 (Invitrogen, US). The second timepoint of monitoring was scheduled at 10 days post-plantation. Diameter and region of interest (ROI) (ImageJ, US) of organoids were measured.

### Statistical Analysis

Correlations between continuous variables were assessed *via* Spearman correlation analysis, while between rank variables were analyzed by Kendall test. The Student t-test, one-way ANOVA, and Pearson’s chi-squared test were used to evaluate differences in variables between groups. The survival probability was analyzed using Kaplan–Meier survival curves and the statistical significance was evaluated by the log-rank test. All statistical analyses were performed using R (version 3.6.1, https://www.r-project.org/). The Bonferroni correction was applied to correct nominal p-values in the subgroup analysis of checkpoint inhibitor immunotherapy reference to Hoshida et al. (43). A P-value < 0.05 was considered statistically significant. All statistical tests were two-sided.

## Results

### Clinical and Molecular Characteristics of PDIA5 in Gliomas

**Figure 1:**
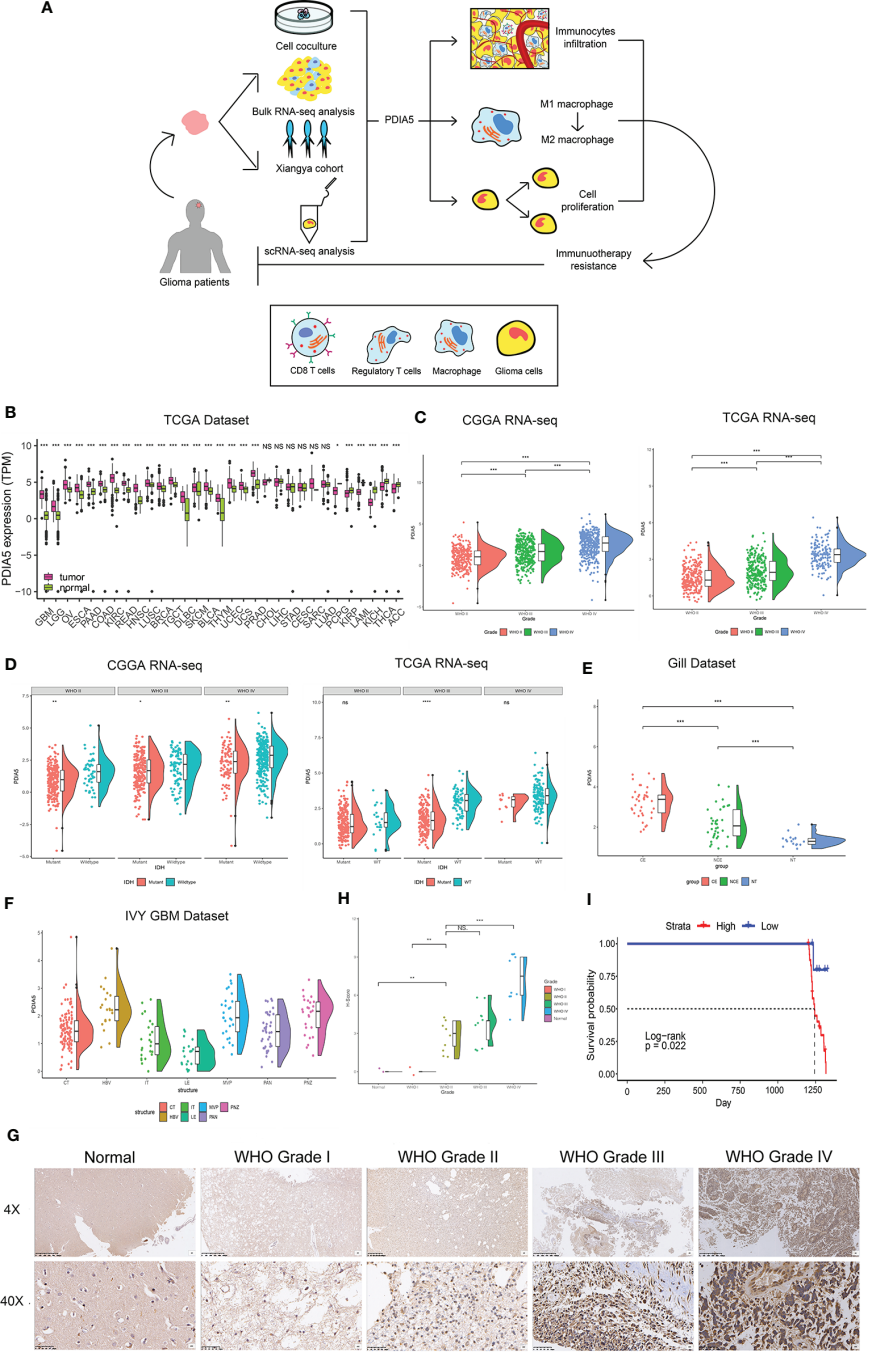
Clinical and molecular characteristics of PDIA5 in gliomas. **(A)** The flow diagram of this research. **(B)** Expression of PDIA5 in multiple human cancers from the The Cancer Genome Atlas (TCGA) dataset. GBM, glioblastoma multiforme; LGG, brain lower grade glioma; OV, ovarian serous cystadenocarcinoma; ESCA, esophageal carcinoma; PAAD, pancreatic adenocarcinoma; COAD, colon adenocarcinoma; KIRC, kidney renal clear cell carcinoma; READ, rectum adenocarcinoma; HNSC, head and neck squamous cell carcinoma; LUSC, lung squamous cell carcinoma; BRCA, breast invasive carcinoma; TGCT, testicular germ cell tumors; DLBC, lymphoid neoplasm diffuse large B-cell lymphoma; SKCM, skin cutaneous melanoma; BLCA, bladder urothelial carcinoma; THYM, thymoma; UCEC, uterine corpus endometrial carcinoma; UCS, uterine carcinosarcoma; PRAD, prostate adenocarcinoma; CHOL, cholangiocarcinoma; LIHC, liver hepatocellular carcinoma; STAD, stomach adenocarcinoma; CESC, cervical squamous cell carcinoma and endocervical adenocarcinoma; SARC, sarcoma; LUAD, lung adenocarcinoma; PCPG, pheochromocytoma and paraganglioma; KIRP, kidney renal papillary cell carcinoma; LAML, acute myeloid leukemia; KICH, kidney chromophobe; THCA, thyroid carcinoma; ACC, adrenocortical carcinoma. **(C)** The expression levels of PDIA5 increased with WHO grade in the Chinese Glioma Genome Atlas (CGGA) and TCGA datasets. **(D)** PDIA5 expression was upregulated in isocitrate dehydrogenase (IDH) wild-type compared with IDH mutant gliomas in CGGA and TCGA datasets. **(E)** PDIA5 expression levels in different radiographical regions of glioblastoma multiforme (GBM) and normal brain from the Gill dataset. **(F)** PDIA5 expression was detected in different locations in the IVY GBM dataset. CT, cellular tumor; HBV, hyperplastic blood vessels; IT, infiltrating tumor; LE, leading edge; MVP, microvascular proliferation; PAN, pseudopalisading cells around necrosis; PNZ, perinecrotic zone. **(G)** Representative images of IHC staining for PDIA5 in normal brain tissue and different WHO grades of glioma [scale bar=625*µm* (upper), 50*µm* (lower)]. **(H)** Quantification (H-score) of PDIA5 IHC staining in normal brain (n=3) and different pathological grades of gliomas (n=31). **(I)** Kaplan-Meier survival curves comparing the high and low expression of PDIA5 in glioma patients from Xiangya Hospital. *P <.05, **P <.01, ***P <.001, ns. p>.05.

The flow diagram of this study was shown in **Figure 1A**. PDIA5 expression in GBM and LGG tissues was higher than in normal tissues (**Figure 1B**). We found no significant correlation between gender and PDIA5 expression in CGGA dataset and TCGA dataset (**Supplementary Figure S1A**), and PDIA5 level was significantly higher in recurrent gliomas and secondary gliomas compared to primary gliomas in the CGGA dataset (**Supplementary Figure S1B**). Additionally, when compared to complete remission/response, PDIA5 expression levels were significantly elevated in patients who experienced progressive disease in response to therapy, whereas no differences were found between other groups (**Supplementary Figure S1C**).

It is well-known that several molecular biomarkers, such as isocitrate dehydrogenase (IDH) mutation, 1p/19q codeletion status and O6-methylguanine DNA methyltransferase (MGMT) promoter methylation are related to the malignancy of gliomas (3, 44). Therefore, these molecular biomarkers were also included into the analysis in addition to WHO grade. PDIA5 expression level was higher in GBM (WHO grade IV) compared to LGG (WHO grade II and grade III) (**Figure 1C**), and it was elevated in malignant histopathologic gliomas (**Supplementary Figure S1D**). In the CGGA dataset, we found that the expression of PDIA5 was higher in the IDH wild-type compared to IDH mutant tumors among different WHO grades (**Figure 1D**). We also found that the PDIA5 expression level was positively associated with 1p/19q non-codeletion status in LGG patients (**Supplementary Figure S1E**). Moreover, PDIA5 expression was upregulated in the MGMT promoter non-methylated samples of pan-glioma patients (**Supplementary Figure S1F**). In summary, our results revealed that PDIA5 expression is upregulated in aggressive gliomas.

Currently, molecular subclasses provides a new perspective to predict disease outcomes (45), and gliomas can be classified into four subtypes: classical (CL), mesenchymal (ME), neural (NE), and pro-neural (PN), among which CL and ME subtypes are more aggressive (46). We detected PDIA5 expression in GBM and pan-gliomas samples from the TCGA dataset and found that increased PDIA5 expression was associated with the CL and ME molecular subtypes (**Supplementary Figure S1G**). Additionally, we evaluated the distribution of PDIA5 expression in GBM and normal tissues using radiographic methods. PDIA5 was found to be highly expressed in control enhanced (CE) regions (**Figure 1E**), which represented tumor cell infiltration. Furthermore, in the IVY GBM dataset, high PDIA5 level was enriched in hyperplastic blood vessels, microvascular proliferation, and peri-necrotic zones compared with other areas (**Figure 1F**).

Protein levels of PDIA5 were examined *via* IHC staining in the gliomas and normal brain tissue samples from Xiangya Hospital (n=34). Demographics and clinical characteristics of these patients are shown in **Supplementary Table S1**. The expression of PDIA5 was higher in GBM (WHO grade IV) compared to LGG (WHO grade II–III) and normal brain tissues (**Figures 1G, H**). Notably, glioma patients with low PDIA5 level experienced favorable outcomes among the glioma patients (**Figure 1I**). These results suggest that PDIA5 is significantly increased in gliomas and high PDIA5 expression may play an important role in invasive processes of gliomas.

### Multifaceted Prognostic Value of PDIA5 in Cancers

**Figure 2:**
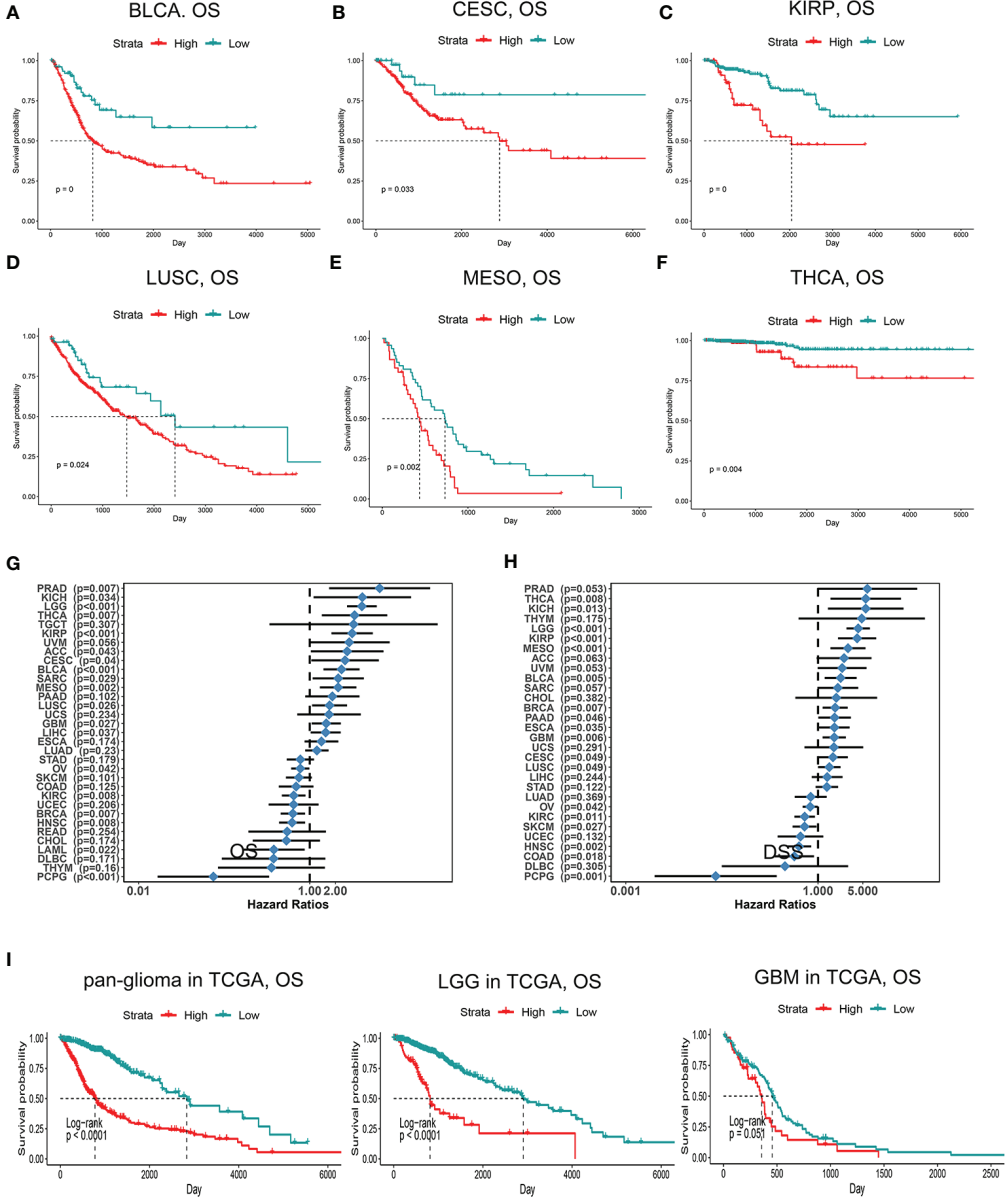
Kaplan-Meier survival curves comparing high and low expression of PDIA5 in different cancers. OS of bladder urothelial carcinoma (BLCA) **(A)**, cervical and endocervical cancers (CESC) **(B)**, kidney renal papillary cell carcinoma (KIRP) **(C)**, lung squamous cell carcinoma (LUSC) **(D)**, mesothelioma (MESO) **(E)**, and thyroid carcinoma (THCA) **(F)**. Correlation of PDIA5 expression with OS **(G)** and DSS **(H)** in 33 types of cancer. OS, overall survival. DSS; disease specific survival. **(I)** Kaplan-Meier analysis of OS based on high *vs.* low expression of PDIA5 in pan-glioma, LGG, and GBM patients in the TCGA dataset. Red curve represents patients with high expression of PDIA5, and blue curve represents low PDIA5.

Since PDIA5 is overexpressed in tumor tissues, we set out to investigate the prognostic value of PDIA5 across cancer types. Patients with high levels of PDIA5 expression experienced shorter OS in bladder urothelial carcinoma (BLCA), cervical and endocervical cancers (CESC), kidney renal papillary cell carcinoma (KIRP), lung squamous cell carcinoma (LUSC), mesothelioma (MESO), and thyroid carcinoma (THCA) (**Figures 2A–G**), and those patients with higher PDIA5 expression levels also experienced shorter DSS (**Figure 2H**, **Supplementary Figures S2A–F**). These findings revealed that high PDIA5 expression predicts poor clinical outcomes in multiple cancers.

We further assessed the prognostic value of PDIA5 in glioma patients from TCGA and CGGA. Among pan-glioma, LGG, and GBM in the TCGA dataset, patients with higher PDIA5 levels presented shorter OS (**Figure 2I**), DSS (**Supplementary Figure S2G**), and PFI (**Supplementary Figure S2H**) compared to patients expressing low levels of PDIA5, with the exception of GBM which was not statistically significant for OS. Similarly, high PDIA5 expression was significantly associated with poor prognosis in the CGGA dataset (**Supplementary Figure S2I**).

Subsequently, we analyzed the effect of PDIA5 on the prognosis of gliomas in the context of different molecular biomarkers and treatments. Regardless of whether IDH was mutated, 1p19q was co-deleted, and MGMT promoter was methylated, low PDIA5 expression was related to a favorable outcome (**Supplementary Figures S3A–F**), and the same results were obtained in the analysis of chemotherapy and radiotherapy (**Supplementary Figures S3G–I**). Analysis of the prognostic significance of PDIA5 in different types of gliomas under the 2016 WHO classification of CNS tumors demonstrated that patients with low PDIA5 expression experienced longer OS regardless of the subtypes (**Supplementary Figures S3J–R**).

### PDIA5 Expression Is Correlated With Distinct Genomic Alterations

To explore the relationship between PDIA5 expression levels and specific genomic alterations in gliomas, CNVs and somatic mutations from the TCGA dataset were analyzed. CNV was investigated between high PDIA5 expression group (n=158) and low PDIA5 expression group (n=158). Amplification of chr7 and deletion of chr10 consistently appeared in gliomas with high PDIA5 expression. Additionally, 1p/19q codeletion more frequently occurred in gliomas with low PDIA5 expression (**Supplementary Figure S4A**), and 63 and 30 significant genomic events were discovered in the high and low PDIA5 groups respectively (**Supplementary Figure S4B**). In the high PDIA5 group, focal amplification peaks, including driver oncogenes such as PIK3C2B (1q32.1), PDGFRA (4q12), EGFR (7p11.2), and CDK4 (12q14.1) were found accompanied by focal deletion peaks for tumor suppressor genes such as CHD5 (1p36.31), CDKN2A/CDKN2B (9p21.3), and PTEN (10q23.31). In the low PDIA5 group, 4q12 amplification peak was observed, but the G score was evidently lower than the high PDIA5 group. Moreover, 19p13.3 amplification peak was also detected, while deletion peaks occurred in 1p32.3, 14q24.2, and 19q13.41. In regards to somatic mutations, mutation in TP53 (41%), TTN (25%), PTEN (23%), and EGFR (22%) were identified in the high PDIA5 group, while IDH1 (89%), CIC (45%), and FUBP1 (22%) were detected in the low PDIA5 group (**Supplementary Figure S4C**).

We also analyzed the correlation between PDIA5 expression and PDIA5 gene copy number, and found that GBM with PDIA5 copy number loss expressed significantly lower levels of PDIA5 mRNA (**Supplementary Figure S5A**). Moreover, in combination analysis of LGG and GBM, we observed PDIA5 expression was higher in the PDIA5 copy number gain group relative to the other two groups (**Supplementary Figure S5B**). These results suggest that PDIA5 expression may be controlled by chromosomal changes in gliomas.

### PDIA5 Is Involved in Immunity Pathways and Inflammatory Activities in Gliomas

**Figure 3:**
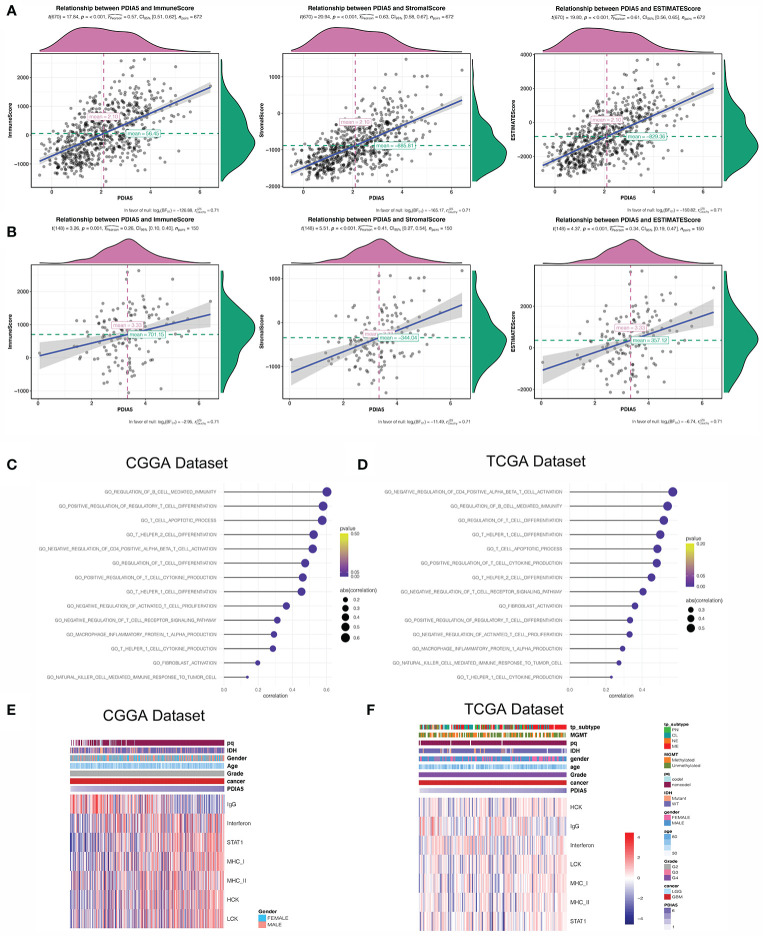
PDIA5 is associated with immunity pathways and inflammatory activities in GBM. PDIA5 expression was positively correlated with immune score, stromal score, and ESTIMATE score in pan-gliomas **(A)** and GBM patients (**B)**. Correlation of PDIA5 and immunity pathways in CGGA **(C)** and TCGA **(D)** datasets. The relationship between PDIA5 and inflammatory activities in the CGGA **(E)** and TCGA **(F)** datasets. Expression values are z-transformed and are highlighted in red for high expression and blue for low expression as indicated in the scale bar.

Previous studies have shown that the extent of immune infiltration in the tumor microenvironment is closely related to prognosis (47), and the aforementioned results support that PDIA5 could be a prognostic signature across cancers. Therefore, we analyzed the correlation between PDIA5 expression and immune infiltration using ESTIMATE, and discovered positive correlation between PDIA5 expression and stromal score, immune score, and ESTIMATE score in pan-glioma (**Figure 3A**) and GBM patients (**Figure 3B**).

Then we continued to conduct correlation analysis between PDIA5 and immunity pathways in gliomas using GO (**Supplementary Table S2**). In GBM patients, PDIA5 was positive associated with regulation of B cell mediated immunity, positive regulation of regulatory T cell differentiation, T cell apoptotic process, T helper2 cell differentiation, negative regulation of CD4 positive alpha beta T cell activation, regulation of T cell differentiation, positive regulation of T cell cytokine production, T helper1 cell differentiation, negative regulation of activated T cell proliferation, negative regulation of T cell receptor signaling pathway, T helper1 cell cytokine production, macrophage inflammatory protein 1 alpha production, fibroblast activation, and natural killer cell mediated immune response to tumor cells in both CGGA and TCGA datasets (**Figures 3C, D**). Similar results were obtained from the analysis of pan-glioma patients (**Supplementary Figures S5C, D**). These findings indicate that PDIA5 may take part in regulating the tumor immune environment of gliomas.

Inflammation response is another essential component of the tumor microenvironment (48). Consequently, we analyzed the association between PDIA5 and seven inflammatory metagenes. PDIA5 expression was positively correlated with interferon, STAT1, MHC-I, MHC-II, HCK and LCH, but negatively correlated with IgG in GBM patients from the CGGA dataset (**Figure 3E**). In the TCGA dataset, PDIA5 was positively correlated with MHC-I, HCK and LCH, and negatively correlated with IgG (**Figure 3F**). Additionally, in pan-glioma patients, there was a positive correlation between PDIA5 expression and six metagenes other than IgG (**Supplementary Figures S5E, F**). These results suggest that PDIA5 is likely to be enriched in signal transduction of T cells and antigen presenting and activation of macrophages, but negatively associated with B lymphocytes in gliomas.

### PDIA5 Is Relevant to Stromal and Immune Cell Infiltration in Gliomas

To investigate the specific mechanism of PDIA5 overexpression promoting immune infiltration, we further explored the correlation between PDIA5 expression and detailed immune cell types in 33 cancer types, and found that PDIA5 was positively correlated with multiple immune cell infiltrates in most cancers including GBM, LGG, and others (**Supplementary Figure S6A**).

**Figure 4:**
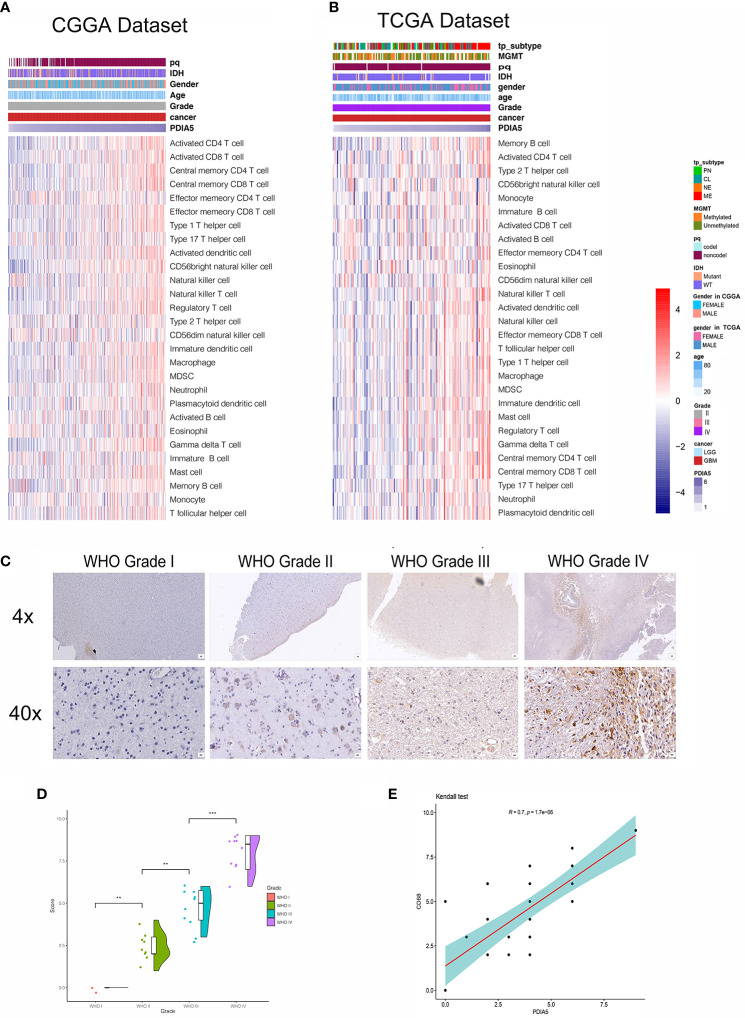
Correlation between PDIA5 expression and immune cell infiltration in gliomas. Correlation of PDIA5 and 28-immune cell lineage genes in glioblastoma multiforme (GBM) from the Chinese Glioma Genome Atlas (CGGA) **(A)** and The Cancer Genome Atlas (TCGA) **(B)** datasets. Expression values are z-transformed and are highlighted in red for high expression and blue for low expression as indicated in the scale bar. **(C)** Representative images of immunohistochemical (IHC) staining for CD68 in different WHO grades of gliomas [scale bar=625*µm* (upper), 50*µm* (lower)]. **(D)** Quantification (H-score) of CD68 IHC staining in normal brain (n=3) and different pathological grades of gliomas (n=31). **(E)** Correlations analysis between PDIA5 and CD68 of IHC staining. **P <.01, ***P <.001.

We then examined the relationship between PDIA5 and 28-immune cell lineage genes in GBM and pan-glioma, and found that the vast majority immune cells, including various types of T cells, B cells, macrophages, myeloid-derived suppressor cells (MDSCs), neutrophils, and natural killer cells, were enriched in the high PDIA5 group of GBM (**Figures 4A, B**) and pan-glioma (**Supplementary Figures S6B, C**). Taken together, these results suggest that high PDIA5 expression level was relevant to stromal and immune cell infiltration in the tumor microenvironment of gliomas.

We also assessed the difference in the expression value of 22 immune cells between high and low expression of PDIA5 group in both CGGA and TCGA dataset using CIBERSORT, and discovered that the differences in macrophages was statistically significant, with M2 macrophages being the most significant (**Supplementary Figures S7A, B**). Positive correlation was also found in the correlation analysis of PDIA5 and macrophage biomarkers (**Supplementary Figures S7C–F**). Consequently, we detected macrophage biomarker CD68 in gliomas and normal brain tissue samples from Xiangya Hospital using IHC staining and found that the number of CD68 positive cells was positively correlated with the WHO grade of gliomas (**Figures 4C, D**). Moreover, a positive relationship was observed in correlation analysis between PDIA5 and CD68 in gliomas patients from TCGA dataset (**Supplementary Figure S8A**). Similarly, the positive correlation between PDIA5 and CD68 was also displayed in the IHC staining samples from Xiangya Hospital (**Figure 4E**). Finally, patients with high PDIA5 and CD68, high combined expression of PDIA5 and CD68 group, and high ratio of PDIA5 to CD68 group experienced shorter OS (**Supplementary Figures S8B–D**). The above findings verify the positive correlation between PDIA5 and macrophage, especially M2, infiltration in gliomas.

### Neoplastic Cells and Macrophages Exhibit High PDIA5 Expression in scRNA-Seq of Gliomas

**Figure 5:**
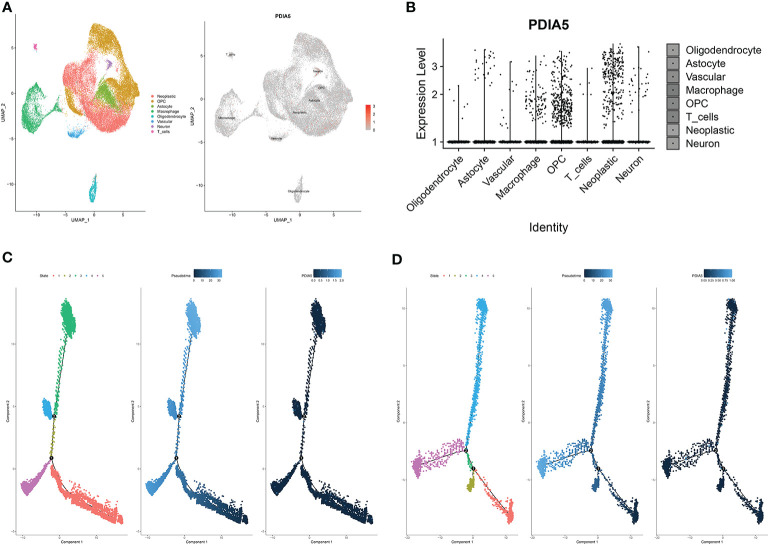
scRNA-seq results for PDIA5 in gliomas. **(A)** The cells were categorized into eight clusters (left). Scatter plots of PDIA5 expression distribution of different cell clusters (right). Gray areas represent the whole cell clusters. The red dots represent cell with PDIA5 expression. **(B)** Violin plot of PDIA5 expression distribution of different cell clusters. **(C)** The single-cell trajectory of neoplastic cells contains four main branches. Cells are colored based on state (left), pseudotime (middle), and PDIA5 (right). **(D)** The single-cell trajectory of macrophages contained four main branches. Cells are colored based on state (left), pseudotime (middle), and PDIA5 (right).

To further elucidate the immune infiltrating role of PDIA5, we also analyzed the expression of PDIA5 in gliomas using scRNA-seq. The representative merged image showing the data from 8 glioma samples is displayed in **Supplementary Figure S8E**. Eight clusters of cells, including neoplastic cells, oligodendrocyte precursor cells (OPCs), astrocytes, macrophages, oligodendrocytes, vascular endothelial cells, neurons, and T cells were identified from the eight glioma samples (**Supplementary Figure S8F**). The expression of PDIA5 in all eight clusters of cells is visualized in **Figure 5A**. We subsequently analyzed the expression level of PDIA5 in the 8 glioma samples. PDIA5 was richly expressed in neoplastic cells, macrophages, and OPCs (**Supplementary Figure S8G**). Additionally, the analysis of the expression level of PDIA5 in different cell clusters further confirmed that PDIA5 was highly correlated with neoplastic cells, macrophages, and OPCs (**Figure 5B**).

We further analyzed the Single-cell pseudotime trajectories and functional annotations of neoplastic cells and macrophages in gliomas. In both neoplastic cells and macrophages, a trajectory was reconstructed by Monocle, which mainly contained two branch points (denoted “1” and “2”) and grouped cells into five states (**Figures 5C, D**). High PDIA5 expression level was observed in state 3 and 4 of neoplastic cells, and particularly higher in state 4 and 5 of macrophages. We further identified 100 genes with branch-dependent expression for branch point 1 of neoplastic cells, the differentially expressed genes before and after branch point 1 and related clustering are visualized in **Supplementary Figure S9A**. The top 12 genes are shown in **Supplementary Figure S9B**. Moreover, 100 differentially expressed genes with branch-dependent expression for branch point 2 of macrophages were also ascertained (**Supplementary Figure S10A**). The top 12 genes are displayed in **Supplementary Figure S10B**. GSEA for neoplastic cells and macrophages for PDIA5 in each “state” is shown in **Supplementary Figures S9C** and **S10C**, respectively. Notably, PDIA5 in state 4 of macrophage was uniformly positively correlated with immune pathways. Finally, the results of GO enrichment analysis (**Supplementary Table S3**, **S4**, **S5**) and KEGG pathway analysis (**Supplementary Table S6**, **S7**, **S8**) in regards to PDIA5 in neoplastic cells and macrophages is shown in **Supplementary Figures S9D–F** and **S10D–F**.

### PDIA5 in Tumor Cells Mediates Tumor Cells Proliferation and Macrophages Exhausting

**Figure 6:**
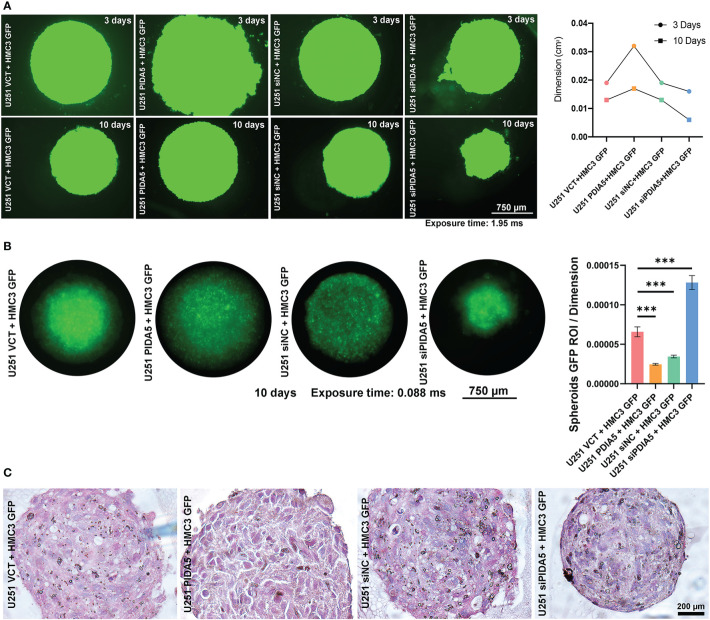
PDIA5 high expression tumor cells exhausted immune cells (HMC3) activation. **(A)** PDIA5 relative U251 lines were co-cultured with HMC3 GFP in organoids medium and monitored and quantified at 3/10 days-post droplets implantation (exposure time: 1.95 ms). **(B)** GFP ROI per dimension measurements valued the viabilities of HMC3 in each co-culturing (exposure time: 0.888 ms). **(C)** HE stained co-cultured organoids demonstrated cell types. Note: figure panel pairs in **(A–C)** represent images captured at differing magnifications; magnification scale bars: panel **(A, B)** *4×amplification: 750 µm;* panel **(C)** *10×amplification: 200 µm*. ***P <.001.

Accumulating evidence of the correlation between PDIA5 and macrophages in glioma microenvironment drove us to investigate the in-depth mechanisms involved in the interconnection among PDIA5, glioma cells, and macrophages. Microglia, the common consensus of residential immune cells in cerebral microenvironment performances essentially as functional macrophages. To investigate PDIA5 functions in glioma, the PDIA5 over-expression plasmid (VCT/PDIA5 plasmid) and siRNA (siNC/siPDIA5) were generated and transfected into U251. Subsequently, PDIA5 relative U251 lines were co-cultured with HMC3 GFP. The dimension of organoid was increased in co-culturing with U251 PDIA5 at 10 days post-transplantation, while dramatically decreased in co-culturing with U251 siPDIA5 (**Figure 6A**). Statistical evaluations of GFP ROIs presenting the HMC3 viabilities was dynamic in co-culturing with U251 siPDIA5 comparing significantly to co-culturing with U251 PDIA5 (**Figure 6B**). Histological sections demonstrated cell types of co-culturing organoids (**Figure 6C**). In co-culturing with U251 siPDIA5, HMC3 was obviously monitored comparing to other organoids. Accordingly, these results demonstrated that PDIA5 high glioma cells functionally promoted tumor cell proliferation and exhausted immune cells (HMC3). Furthermore, knock-down PDIA5 presented the malignant behavior decreasing of glioma cells in immune cells exhausting.

### Immunotherapy Is More Practical for High PDIA5 Patients

**Figure 7:**
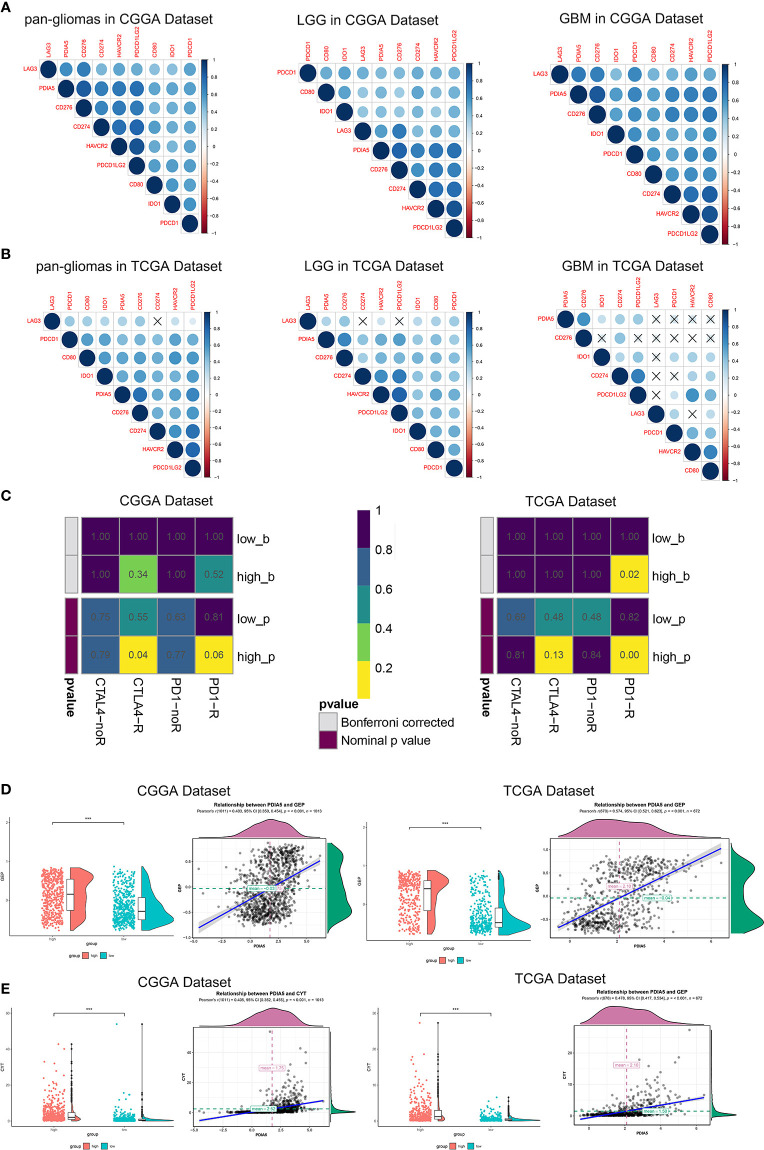
Immunotherapy is more practical for high PDIA5 patients. Correlation between PDIA5 and immune checkpoint members in pan-gliomas, low grade glioma (LGG), and glioblastoma multiforme (GBM) from the Chinese Glioma Genome Atlas (CGGA) **(A)** and The Cancer Genome Atlas (TCGA) **(B)** datasets. **(C)** Submap analysis of the response of anti-CTLA-4 and anti-PD-1 therapy in the CGGA and TCGA datasets. low_b, or high_b was the value obtained from low_p, or high_p multiplied by 8 (2 *4) based on Bonferroni correction, respectively. **(D)** The relationship between PDIA5 and T cell-inflamed gene expression profile (GEP) level in the CGGA and TCGA datasets. **(E)** The relationship between PDIA5 and cytolytic activity (CYT) in the CGGA and TCGA datasets. ***P <.001.

In recent years, immunotherapies, which target the immune checkpoint molecules including CTLA-4, PD-1, and PD-L1/2 have offered new treatment opportunities and improved survival in hard-to-treat tumors (7, 8). To assess the correlation between PDIA5 and immune checkpoints, we selected several well-known immune checkpoints, including LAG3, HAVCR2 (TIM-3), CD274 (PD-L1), CD276 (B7-H3), CD80, PDCD1LG2 (PD-L2), PDCD1 (PD-1), and IDO1 for correlation analysis. In CGGA dataset, PDIA5 expression was positively associated with CD276, CD274, PDCD1LG2, and HAVCR2 in pan-glioma and LGG patients, and positively correlated with CD276, PDCD1, CD274, PDCD1LG2, and HAVCR2 in GBM patients. In TCGA datasets, there was positive correlation between PDIA5 and CD276 or PDCD1LG2 in pan-glioma patients, and PDIA5 expression was positively associated with CD276, PDCD1LG2 and HAVCR2 in LGG patients. However, in GBM patients, PDIA5 only demonstrated a strong positive correlation with CD276 (**Figures 7A, B**). Altogether, our results imply that PDIA5 has positive correlation with clinically relevant immune checkpoint molecules in gliomas.

Subsequently, we investigated whether PDIA5 could predict glioma patients’ responses to checkpoint inhibitor immunotherapy in anti-CTLA-4 and anti-PD-1 based on CGGA and TCGA datasets, and found that compared with low PDIA5 group, high PDIA5 group was expected to respond better to immunotherapies (**Figure 7C**). Previous work indicates that GEP and CYT are able to enhance anti-tumor activity and associated with the response to PD-1 inhibitor (49, 50). Therefore, we further explored the relationship between PDIA5 and GEP as well as CYT. PDIA5 was found to be positively associated with GEP and CYT (**Figures 7D, E**).

**Figure 8:**
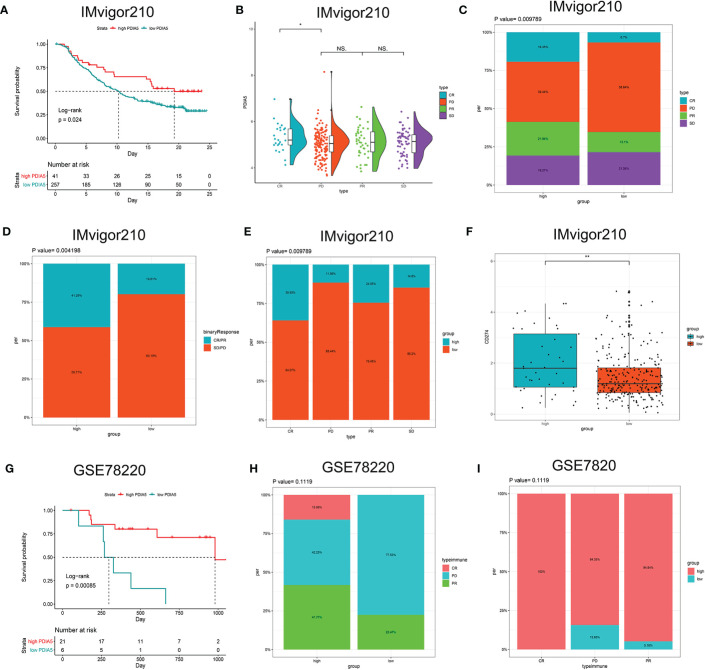
The role of PDIA5 in predicting the therapeutic value of checkpoint blockade immunotherapy. **(A)** Kaplan–Meier survival plot showed a significant survival benefit in the high PDIA5 group of IMvigor210 cohort. **(B)** Distribution of PDIA5 in the distinct anti-PD-L1 clinical response group. *p <.05, ns, p >.05. **(C)** The proportions of clinical response to anti-PD-L1 immunotherapy in the high and low PDIA5 groups. **(D)** The proportions of clinical binary response to anti-PD-L1 immunotherapy in the high and low PDIA5 groups. **(E)** The proportions of the high and low PDIA5 groups in the anti-PD-L1 immunotherapy clinical response. **(F)** Differences in CD274 (PD-L1) expression in the high and low PDIA5 groups in the IMvigor210 cohort. **(G)** Kaplan–Meier survival plot showed a significant survival benefit in the high PDIA5 group of GSE78220 cohort. **(H)** The proportions of clinical response to anti-PD-1 immunotherapy in the high and low PDIA5 groups. **(I)** The proportions of the high and low PDIA5 groups in the anti-PD-1 immunotherapy clinical response. **p < .01.

Then we continued to analyze the predictive value of PDIA5 regarding the response of anti-PD-L1 (IMvigor210) and anti-PD-1 (GSE78220) therapy for urothelial cancer and metastatic melanoma cohorts, respectively. In the anti-PD-L1 cohort (IMvigor210), we observed that patients with high PDIA5 experienced significant clinical survival benefits (**Figure 8A**). The significant treatment strengths and response to anti-PD-L1 immunotherapy in high PDIA5 group compared to the low PDIA5 group were also verified (**Figures 8B–E**). In the anti-PD-L1 cohort, the percentages of complete response (CR) and progressive disease (PD) were 19.35 and 39.44% in the high PDIA5 group, respectively, and 6.7 and 58.64% in the low PDIA5 group, respectively. And the proportion of high PDIA5 expression in CR group and PD group were 35.93 and 11.56%, respectively. Additionally, the high PDIA5 group exhibited high expression of CD274 (PD-L1), which resulted in good response to anti-PD-L1 therapy (**Figure 8F**). Similarly, notable favorable outcome of the high PDIA5 group was also observed in the anti-PD-1 cohort (GSE78220) (**Figure 8G**). The frequencies of CR, PD, and partial response (PR) were 15.99, 42.25, and 41.77% in the high PDIA5 group, respectively, and 0%, 77.53, and 22.47% in the low PDIA5 group, respectively (**Figure 8H**). And the proportion of high PDIA5 expression in CR group, PD group, and PR group were 100, 84.35, and 94.84%, respectively (**Figure 8I**). The difference is not statistically significant possibly due to the small sample size. The above findings suggest that patients with high PDIA5 have high anti-tumor immune activity and may benefit from immunotherapies.

## Discussion

Based on large-scale bioinformatic analysis, we are the first to comprehensively analyze PDIA5 expression profiles in gliomas according to the WHO grading system, histopathology, molecular biomarkers, and molecular subclasses. PDIA5 expression levels were elevated in malignant gliomas ground on the above different categories. PDIA5 overexpression was also found in the areas of infiltrating tumor cells according to radiology imaging. Importantly, our results show that high levels of PDIA5 expression predict poor outcomes based on survival analysis of different subgroups.

Genomic alterations in gliomas are able to predict disease classification and prognosis (51). In CNV analysis, we found focal amplification peaks for oncogenes in the high PDIA5 group and focal deletion peaks for tumor suppressor genes. Several common somatic mutations in GBM including TP53, TTN, PTEN, and EGFR (52), were also present in the high PDIA5 group. These results suggest that high PDIA5 expression plays an important role in glioma infiltration. Investigating the detailed mechanism of PDIA5 promotion of glioma development may help to develop new therapeutic strategies.

High-grade gliomas progress rapidly, and cause short survival of patients, among which GBM harbors the most severe malignancy. The role of the immune microenvironment in the progression of gliomas has become increasingly well-known (53). Previous research has shown that the tumor immune microenvironment influences gene expression of tumor tissues and the degree of stromal and immune cell infiltration contribute notably to prognosis (54). Stromal score, immune score, and ESTIMATE score, which are based on ESTIMATE algorithm, were shown to be negatively correlated with the prognosis of GBM (47), glioma, oligodendroglioma, melanoma (55), and gastric cancer (56). In our research, we found these three ESTIMATE algorithm scores were increased along with PDIA5, indicating that high expression level of PDIA5 is positively correlated with immune infiltration in gliomas.

Infiltrating immune cells in glioma tumor microenvironment are comprised of microglia/macrophages, CD4+ T cells, regulatory T cells (Tregs), MDSCs, and granulocytes, among which microglia and MDSC are the most frequent (53), contributing to ineffective immune activation in GBM (57). Our results revealed that multiple immune cell types were enriched in high PDIA5 patients. And the correlation between PDIA5 and T cells as well as macrophages in gliomas was presented in the subsequent specific analysis.

So far, no previous studies have focused on the interaction between PDIA5 and tumor immunity, but a few studies on PDI and immune cells have demonstrated that PDI can elicit CD8+ T-cells in leishmaniasis (58, 59). Caorsi et al. have shown PDIA3 induced proliferation of autologous CD4 and CD8 T cells in colorectal cancer, accompanied by PDIA3-specific Th1 effector cell accumulation in tumor tissue (60). Besides, the other member of PDI family has also been identified to be relevant to the activation and function of macrophages (61). Most of the functions of PDIA5 remains unclear, but the b-type domain of PDIA5 has a binding region for PDIA3 (62), suggesting the possibility that PDIA5 may be related to some biological functions of PDIA3. However, basic research is needed to further investigate the specific interactions between PDIA5 and the immune system in gliomas.

Gliomas, especially GBM, can escape anti-tumor immunity and cause severe T cell dysfunction, which includes the apoptosis of effector T cells and the activation of Tregs (63). Our correlation analysis of immune pathways showed that PDIA5 was positively associated with the differentiation of regulatory T cell and the apoptotic process of T cells, while negative correlated with CD4+ T cell activation and proliferation. Besides, the investigation about inflammatory activity suggested that PDIA5 was also enriched in the biological process of T cells. The above indicating that PDIA5 may be associated with abnormal T cell function in gliomas.

Additionally, growing evidence have identified that tumor-associated macrophages (TAMs) played a key role in the progression and metastasis of tumor cells (64–66). As the result of the impact of metabolites of malignant cells, TAMs in the tumor microenvironment make corresponding metabolic changes, leading to functional reprogramming of TAMs which includes the M2 polarization of macrophages, and alterations of cytokines and angiogenic factors secretion. These above changes are conducive to the migration and invasion of tumors (67, 68). Our immunohistochemistry results found that the number of macrophages increased with the WHO grade of gliomas, which was consistent with previous studies. Further analysis attested the positive correlation between PDIA5 and macrophage, especially M2, infiltration in gliomas. Therefore, we deduced that high expression of PDIA5 may induce macrophage associated immunity, and contribute to M2 polarization of macrophage in gliomas.

To learn more about the role of PDIA5 in macrophage associated immunity and malignant cell proliferation of gliomas, scRNA-seq analysis and gain of function as well as loss of function assay were performed. To date, scRNA-seq has exhibited great potential in screening therapeutic targets for antitumor immunity. Several studies have analyzed the gene expression of immune cells in gliomas using scRNA-seq data. Goswami et al. identified CD73 as a specific immunotherapy target which enhances the antitumor immune response to immune checkpoint therapy in GBM using scRNA-seq (69), and Cheng et al. identified 31 genes that could be biomarkers for GBM tumor cells based on single cell sequencing (70). Additionally, pseudotime trajectories analysis is capable of capturing and dissecting transcriptional changes in cells during glioma progression. Therefore, it can be used to evaluate the relationship between genes and development of specific cell lineages in gliomas (41). In the present study, using scRNA-seq data, we found that high PDIA5 expression existed in neoplastic cells and macrophages of gliomas, further research on pseudotime trajectories and functional annotations emphasized the correlation between PDIA5 and macrophage infiltration as well as progression in gliomas. Moreover, cell transfection and co-culture of glioma cells and macrophages based on organoids revealed that PDIA5 in tumor cells mediated glioma cells proliferation and macrophages exhausting, which further confirmed the crucial role of PDIA5 in regulating immune activity in the tumor microenvironment of glioma.

Nevertheless, the overexpression of PDIA5 resulted in the ultimate exhaustion of macrophage in our *in vitro* experiments, which was contrary to the findings that PDIA5 was positively correlated with macrophage infiltration in our previous bioinformatic analysis. This phenomenon can be attributed to the fact that the glioma cells with high expression of PDIA5 secrete certain cytokines to recruit M2 macrophages, which interact with glioma cells (67) and potentially ended with apoptosis and degradation. And the consumed macrophages can be continuously replenished from the peripheral blood and resident microglia in brain *in vivo* (68), while the number of macrophages in the co-culture system is constant, eventually led to the exhaustion of macrophages. Therefore, the high level of PDIA5 expression in gliomas indeed contribute to recruiting macrophages and probably mediating the polarization of macrophages to M2. Taken together, our findings demonstrate that PDIA5 overexpression correlates with immune infiltration and inflammation in gliomas, which may lead to poor prognosis in glioma patients.

Immune checkpoint refers to specific molecular interactions at the interface between T cells and antigen presenting cells, and exhibits the ability to inhibition T cell function (63). Targeting immune checkpoint, which enhances anti-tumor immune responses, has brought about remarkable clinical advances and offered new targets for tumor therapy (7). Each immune checkpoint has its own unique molecular characteristics, and several immune checkpoints may interact with each other. The prominent PD-1/PD-L1 axis, can promote invasion of GBM cells in brain tissue (71). Additionally, PD-1 has also proven to be correlated with other immune checkpoints including IDO1, LAG3, TIM-3, and B7-H3 (72). Li et al. found that glioma patients had higher TIM-3 expression on peripheral innate immunocytes, which further contributed to immune disorders (73). B7H3 has been reported to play a pivotal role in cell differentiation and carcinogenesis of glioma by Zhang et al. (74), And PD-L2, another ligand of PD-1, can evade antitumor immunity through modulating T cell response and proliferation in gliomas (75). We found tight correlations between PDIA5 and B7-H3, PD-L2, and TIM-3, suggesting that PDIA5 probably plays a synergistic role with those immune checkpoints in the progression of glioma. Further predictive analysis based on the existing databases showed that patients with high PDIA5 had high anti-tumor immune activity and were more likely to benefit from immunotherapies in gliomas as well as other tumor types, indicating that inhibition of combined PDIA5 and these immune checkpoints could improve the clinical management of gliomas.

Hsowever, there are still some limitations regarding this study, which are expected to be improved on in subsequent studies. Firstly, the relationship between PDIA5 and B cells is unclear or even seems to be contradictory in different analyses relative to macrophages and T cells. Despite the negative association between PDIA5 and IgG indicate PDIA5 inhibition of IgG activity, which might only represent part of the B cells, PDIA5 promotion of malignancy attract more immune cells including B cells in the tumor microenvironment of gliomas. Secondly, the high anti-tumor activity and poor clinical outcomes are another discrepancy among patients with high PDIA5. Prior evidence implicated that high PD-L1 contributed to immunosuppression but enhanced the response rate to anti-PD-1 therapy in metastatic melanomas and breast cancer (76, 77). The authors suggested that pre-treatment high level of PD-L1 may be related to its role in immune dysfunction and T cell exhaustion, while the increased PD-L1 level in on-treatment patients was caused by the reinvigoration of T cells. And the loss of PD-L1 inhibition effect on T cells was due to the interaction between PD-L1 and PD-1 blocked by anti-PD-1 therapy. Similar interplay patterns potentially exist between PDIA5 and certain immune checkpoint, like PD-1 and CTLA-4, giving rise to better responses to checkpoint inhibitor immunotherapy. Generally, existing data regarding the action mechanism of PDIA5 to interfere with the immune system is relatively lacking, and more wet experiments are needed to further interpret the role of PDIA5 in gliomas immunology.

In summary, our findings demonstrate that PDIA5 is upregulated in multiple types of malignant gliomas, and has multifaceted prognostic value in cancers. It is particularly noteworthy that PDIA5 overexpression correlates with immune infiltration and is associated with poor prognosis in glioma patients. And patients with high PDIA5 are more likely to benefit from immunotherapies. Overall, these findings indicate that PDIA5 could be a promising target for glioma immunotherapy.

## Data Availability Statement

The datasets analyzed during the current study are available in the Gene Expression Omnibus (https://www.ncbi.nlm.nih.gov/geo/), TCGA data source (https://xena.ucsc.edu) and CGGA data portal (http://www.cgga.org.cn).

## Ethics Statement

The studies involving human participants were reviewed and approved by the Ethics Committee of Xiangya Hospital, Central South University. The patients/participants provided their written informed consent to participate in this study.

## Author Contributions

HZ and JH designed the study and interpreted data. QC and LZ provided foundation support and supervised the study. HZ, JH, ZD, and ZW acquired and analyzed data. HZ, JH, QC, HC, ZX, FH, and SF drafted the manuscript and revised for submission quality. All authors contributed to the article and approved the submitted version.

## Funding

This work was supported by the National Natural Science Foundation of China (No. 82073893, No.81703622, No.81402249, No.81903015); China Postdoctoral Science Foundation (No.2018M633002); Natural Science Foundation of Hunan Province (No.2018JJ3838, No. S2019JJQNJJ1625); Hunan Provincial Health and Health Committee Foundation of China (C2019186); and Xiangya Hospital Central South University postdoctoral foundation.

## Conflict of Interest

The authors declare that the research was conducted in the absence of any commercial or financial relationships that could be construed as a potential conflict of interest.

## Abbreviations

GBM, glioblastoma multiforme; WHO, World Health Organization; CNS, central nervous system; PDI, protein disulfide isomerase; ER, endoplasmic reticulum; PDIA5, protein disulfide isomerase A5; PDIR, protein disulfide isomerase-related protein; UPR, unfolded protein response; CGGA, Chinese Glioma Genome Atlas; TCGA, The Cancer Genome Atlas; GEO, Gene Expression Omnibus; LGG, low grade glioma; IHC, immunohistochemical; scRNA-seq, single-cell RNA sequencing; OS, overall survival; DSS, disease specific survival; HRs, hazard ratios; CI, confidence intervals; PFI, progression-free interval; CNVs, somatic copy variations; ESTIMATE, Estimation of Stromal and Immune cells in Malignant Tumor tissues using Expression; GSVA, gene set variation analysis; GEP, gene expression profile; CYT, cytolytic activity; PCA, principal component analysis; GSEA, gene set enrichment analysis; GO, gene ontology; KEGG, Kyoto Encyclopedia of Genes and Genomes; ROI, region of interest; IDH, isocitrate dehydrogenase; MGMT, O6-methylguanine DNA methyltransferase; CL, classical; ME, mesenchymal; NE, neural; PN, proneural; CE, control enhanced; BLCA, bladder urothelial carcinoma; CESC, cervical and endocervical cancers; KIRP, kidney renal papillary cell carcinoma; LUSC, lung squamous cell carcinoma; MESO, mesothelioma; THCA, thyroid carcinoma; Treg, regulatory T cells; MDSCs, myeloid-derived suppressor cells; OPCs, oligodendrocyte precursor cells.

## References

[1] Weller M Wick W Aldape K Brada M Berger M Pfister SM. Glioma. Nat Rev Dis Primers (2015) 1:15017. 10.1038/nrdp.2015.17 27188790

[2] Zhang H Wang R Yu Y Liu J Luo T Fan F. Glioblastoma Treatment Modalities besides Surgery. J Cancer (2019) 10(20):4793–806. 10.7150/jca.32475 PMC6775524 31598150

[3] Jiang T Mao Y Ma W Mao Q You Y Yang X. CGCG clinical practice guidelines for the management of adult diffuse gliomas. Cancer Lett (2016) 375(2):263–73. 10.1016/j.canlet.2016.01.024 26966000

[4] Zeng F Wang K Liu X Zhao Z. Comprehensive profiling identifies a novel signature with robust predictive value and reveals the potential drug resistance mechanism in glioma. Cell Commun Signal (2020) 18(1):2. 10.1186/s12964-019-0492-6 31907037 PMC6943920

[5] Audia A Conroy S Glass R Bhat KPL. The Impact of the Tumor Microenvironment on the Properties of Glioma Stem-Like Cells. Front Oncol (2017) 7:143. 10.3389/fonc.2017.00143 28740831 PMC5502267

[6] Ma Q Long W Xing C Chu J Luo M Wang HY. Cancer Stem Cells and Immunosuppressive Microenvironment in Glioma. Front Immunol (2018) 9:2924. 10.3389/fimmu.2018.02924 30619286 PMC6308128

[7] Romani M Pistillo MP Carosio R Morabito A Banelli B. Immune Checkpoints and Innovative Therapies in Glioblastoma. Front Oncol (2018) 8:464. 10.3389/fonc.2018.00464 30406030 PMC6206227

[8] Sharma P Allison JP. The future of immune checkpoint therapy. Science (2015) 348(6230):56–61. 10.1126/science.aaa8172 25838373

[9] Hodges TR Ott M Xiu J Gatalica Z Swensen J Zhou S. Mutational burden, immune checkpoint expression, and mismatch repair in glioma: implications for immune checkpoint immunotherapy. Neuro Oncol (2017) 19(8):1047–57. 10.1093/neuonc/nox026 PMC5570198 28371827

[10] Gibellini L De Biasi S Porta C Lo Tartaro D Depenni R Pellacani G. Single-Cell Approaches to Profile the Response to Immune Checkpoint Inhibitors. Front Immunol (2020) 11:490. 10.3389/fimmu.2020.00490 32265933 PMC7100547

[11] Louis DN Perry A Reifenberger G von Deimling A Figarella-Branger D Cavenee WK. The 2016 World Health Organization Classification of Tumors of the Central Nervous System: a summary. Acta Neuropathol (2016) 131(6):803–20. 10.1007/s00401-016-1545-1 27157931

[12] Xu S Liu Y Yang K Wang H Shergalis A Kyani A. Inhibition of protein disulfide isomerase in glioblastoma causes marked downregulation of DNA repair and DNA damage response genes. Theranostics (2019) 9(8):2282–98. 10.7150/thno.30621 PMC6531306 31149044

[13] Ferrari DM Söling HD. The protein disulphide-isomerase family: unravelling a string of folds. Biochem J (1999) 339(Pt 1):1–10. 10.1042/0264-6021:3390001 10085220 PMC1220120

[14] Galligan JJ Petersen DR. The human protein disulfide isomerase gene family. Hum Genomics (2012) 6:6. 10.1186/1479-7364-6-6 23245351 PMC3500226

[15] Wang Z Zhang H Cheng Q. PDIA4: The basic characteristics, functions and its potential connection with cancer. BioMed Pharmacother (2020) 122:109688. 10.1016/j.biopha.2019.109688 31794946

[16] Peng Z Chen Y Cao H Zou H Wan X Zeng W. Protein disulfide isomerases are promising targets for predicting the survival and tumor progression in glioma patients. Aging (Albany NY) (2020) 12(3):2347–72. 10.18632/aging.102748 PMC7041756 32023222

[17] Horibe T Torisawa A Masuda Y Kawakami K. Functional analysis of protein disulfide isomerase P5 in glioblastoma cells as a novel anticancer target. Oncol Rep (2019) 41(2):961–72. 10.3892/or.2018.6868 30431130

[18] Ramos FS Serino LT Carvalho CM Lima RS Urban CA Cavalli IJ. PDIA3 and PDIA6 gene expression as an aggressiveness marker in primary ductal breast cancer. Genet Mol Res (2015) 14(2):6960–7. 10.4238/2015.June.26.4 26125904

[19] Tufo G Jones AWE Wang Z Hamelin J Tajeddine N Esposti DD. The protein disulfide isomerases PDIA4 and PDIA6 mediate resistance to cisplatin-induced cell death in lung adenocarcinoma. Cell Death Differ (2014) 21(5):685–95. 10.1038/cdd.2013.193 PMC3978299 24464223

[20] Silva Z Verissimo T Videira PA Novo C. Protein disulfide isomerases: Impact of thapsigargin treatment on their expression in melanoma cell lines. Int J Biol Macromol (2015) 79:44–8. 10.1016/j.ijbiomac.2015.04.029 25912252

[21] Xu S Sankar S Neamati N. Protein disulfide isomerase: a promising target for cancer therapy. Drug Discov Today (2014) 19(3):222–40. 10.1016/j.drudis.2013.10.017 24184531

[22] Lee E Lee D. Emerging Roles of Protein Disulfide Isomerase in Cancer. BMB Rep (2017) 50(8):401–10. 10.5483/bmbrep.2017.50.8.107 PMC5595169 28648146

[23] Hu Q Huang K Tao C Zhu X. Protein disulphide isomerase can predict the clinical prognostic value and contribute to malignant progression in gliomas. J Cell Mol Med (2020) 24(10):5888–900. 10.1111/jcmm.15264 PMC7214159 32301283

[24] Zou H Wen C Peng Z Shao Y Hu L Li S. P4HB and PDIA3 are associated with tumor progression and therapeutic outcome of diffuse gliomas. Oncol Rep (2018) 39(2):501–10. 10.3892/or.2017.6134 PMC5783617 29207176

[25] Sun S Lee D Ho AS Pu JK Zhang XQ Lee NP. Inhibition of prolyl 4-hydroxylase, beta polypeptide (P4HB) attenuates temozolomide resistance in malignant glioma via the endoplasmic reticulum stress response (ERSR) pathways. Neuro Oncol (2013) 15(5):562–77. 10.1093/neuonc/not005 PMC3635523 23444257

[26] Hayano T Kikuchi M. Molecular cloning of the cDNA encoding a novel protein disulfide isomerase-related protein (PDIR). FEBS Lett (1995) 372(2-3):210–4. 10.1016/0014-5793(95)00996-m 7556671

[27] Kuo TF Chen TY Jiang ST Chen KW Chiang YM Hsu YJ. Protein disulfide isomerase a4 acts as a novel regulator of cancer growth through the procaspase pathway. Oncogene (2017) 36(39):5484–96. 10.1038/onc.2017.156 28534513

[28] Higa A Taouji S Lhomond S Jensen D Fernandez-Zapico ME Simpson JC. Endoplasmic reticulum stress-activated transcription factor ATF6α requires the disulfide isomerase PDIA5 to modulate chemoresistance. Mol Cell Biol (2014) 34(10):1839–49. 10.1128/MCB.01484-13 PMC4019026 24636989

[29] Hurst KE Lawrence KA Reyes Angeles L Ye Z Zhang J Townsend DM. Endoplasmic Reticulum Protein Disulfide Isomerase Shapes T Cell Efficacy for Adoptive Cellular Therapy of Tumors. Cells (2019) 8(12):1514. 10.3390/cells8121514 PMC6953024 31779147

[30] Gill BJ Pisapia DJ Malone HR Goldstein H Lei L Sonabend A. MRI-localized biopsies reveal subtype-specific differences in molecular and cellular composition at the margins of glioblastoma. Proc Natl Acad Sci U S A (2014) 111(34):12550–5. 10.1073/pnas.1405839111 PMC4151734 25114226

[31] Wang L Babikir H Müller S Yagnik G Shamardani K Catalan F. The Phenotypes of Proliferating Glioblastoma Cells Reside on a Single Axis of Variation. Cancer Discov (2019) 9(12):1708–19. 10.1158/2159-8290.cd-19-0329 PMC7161589 31554641

[32] Mariathasan S Turley SJ Nickles D Castiglioni A Yuen K Wang Y. TGFbeta attenuates tumour response to PD-L1 blockade by contributing to exclusion of T cells. Nature (2018) 554(7693):544–8. 10.1038/nature25501 PMC6028240 29443960

[33] Hugo W Zaretsky JM Sun L Song C Moreno BH Hu-Lieskovan S. Genomic and Transcriptomic Features of Response to Anti-PD-1 Therapy in Metastatic Melanoma. Cell (2016) 165(1):35–44. 10.1016/j.cell.2016.02.065 26997480 PMC4808437

[34] Ni M Liu X Wu J Zhang D Tian J Wang T. Identification of Candidate Biomarkers Correlated With the Pathogenesis and Prognosis of Non-small Cell Lung Cancer via Integrated Bioinformatics Analysis. Front Genet (2018) 9:469. 10.3389/fgene.2018.00469 30369945 PMC6194157

[35] Mermel CH Schumacher SE Hill B Meyerson ML Beroukhim R Getz G. GISTIC2.0 facilitates sensitive and confident localization of the targets of focal somatic copy-number alteration in human cancers. Genome Biol (2011) 12(4):R41. 10.1186/gb-2011-12-4-r41 21527027 PMC3218867

[36] Yoshihara K Shahmoradgoli M Martínez E Vegesna R Kim H Torres-Garcia W. Inferring tumour purity and stromal and immune cell admixture from expression data. Nat Commun (2013) 4(1):2612. 10.1038/ncomms3612 24113773 PMC3826632

[37] Hänzelmann S Castelo R Guinney J. GSVA: gene set variation analysis for microarray and RNA-seq data. BMC Bioinformatics (2013) 14:7. 10.1186/1471-2105-14-7 23323831 PMC3618321

[38] Ye Y Jing Y Li L Mills GB Diao L Liu H. Sex-associated molecular differences for cancer immunotherapy. Nat Commun (2020) 11(1):1779. 10.1038/s41467-020-15679-x 32286310 PMC7156379

[39] Stuart T Butler A Hoffman P Hafemeister C Papalexi E Mauck III WM. Comprehensive Integration of Single-Cell Data. Cell (2019) 177(7):1888–902.e21. 10.1016/j.cell.2019.05.031 31178118 PMC6687398

[40] Aran D Looney AP Liu L Wu E Fong V Hsu A. Reference-based analysis of lung single-cell sequencing reveals a transitional profibrotic macrophage. Nat Immunol (2019) 20(2):163–72. 10.1038/s41590-018-0276-y PMC6340744 30643263

[41] Pang B Xu J Hu J Guo F Wan L Cheng M. Single-cell RNA-seq reveals the invasive trajectory and molecular cascades underlying glioblastoma progression. Mol Oncol (2019) 13(12):2588–603. 10.1002/1878-0261.12569 PMC6887585 31487431

[42] Xu J Zhang Z Qian M Wang S Qiu W Chen Z. Cullin-7 (CUL7) is overexpressed in glioma cells and promotes tumorigenesis via NF-kappaB activation. J Exp Clin Cancer Res (2020) 39(1):59. 10.1186/s13046-020-01553-7 32252802 PMC7132976

[43] Hoshida Y Brunet JP Tamayo P Golub TR Mesirov JP. Subclass mapping: identifying common subtypes in independent disease data sets. PloS One (2007) 2(11):e1195. 10.1371/journal.pone.0001195 18030330 PMC2065909

[44] Eckel-Passow JE Lachance DH Molinaro AM Walsh KM Decker PA Sicotte H. Glioma Groups Based on 1p/19q,IDH, andTERTPromoter Mutations in Tumors. N Engl J Med (2015) 372(26):2499–508. 10.1056/NEJMoa1407279 PMC4489704 26061753

[45] Verhaak RGW Hoadley KA Purdom E Wang V Qi Y Wilkerson MD. Integrated Genomic Analysis Identifies Clinically Relevant Subtypes of Glioblastoma Characterized by Abnormalities in PDGFRA, IDH1, EGFR, and NF1. Cancer Cell (2010) 17(1):98–110. 10.1016/j.ccr.2009.12.020 20129251 PMC2818769

[46] Phillips HS Kharbanda S Chen R Forrest WF Soriano RH Wu TD. Molecular subclasses of high-grade glioma predict prognosis, delineate a pattern of disease progression, and resemble stages in neurogenesis. Cancer Cell (2006) 9(3):157–73. 10.1016/j.ccr.2006.02.019 16530701

[47] Jia D Li S Li D Xue H Yang D Liu Y. Mining TCGA database for genes of prognostic value in glioblastoma microenvironment. Aging (Albany NY) (2018) 10(4):592–605. 10.18632/aging.101415 29676997 PMC5940130

[48] Lepore F D’Alessandro G Antonangeli F Santoro A Esposito V Limatola C. CXCL16/CXCR6 Axis Drives Microglia/Macrophages Phenotype in Physiological Conditions and Plays a Crucial Role in Glioma. Front Immunol (2018) 9:2750. 10.3389/fimmu.2018.02750 30542347 PMC6277753

[49] Ayers M Lunceford J Nebozhyn M Murphy E Loboda A Kaufman DR. IFN-gamma-related mRNA profile predicts clinical response to PD-1 blockade. J Clin Invest (2017) 127(8):2930–40. 10.1172/JCI91190 PMC5531419 28650338

[50] Rooney MS Shukla SA Wu CJ Getz G Hacohen N. Molecular and genetic properties of tumors associated with local immune cytolytic activity. Cell (2015) 160(1-2):48–61. 10.1016/j.cell.2014.12.033 25594174 PMC4856474

[51] McNulty SN Cottrell CE Vigh-Conrad KA Carter JH Heusel JW Ansstas G. Beyond sequence variation: assessment of copy number variation in adult glioblastoma through targeted tumor somatic profiling. Hum Pathol (2019) 86:170–81. 10.1016/j.humpath.2018.12.004 30594748

[52] Segura-Collar B Gargini R Tovar-Ambel E Hernandez-SanMiguel E Epifano C Perez de Castro I. The EGFR-TMEM167A-p53 Axis Defines the Aggressiveness of Gliomas. Cancers (Basel) (2020) 12(1):208. 10.3390/cancers12010208 PMC7017250 31947645

[53] Gieryng A Pszczolkowska D Walentynowicz KA Rajan WD Kaminska B. Immune microenvironment of gliomas. Lab Invest (2017) 97(5):498–518. 10.1038/labinvest.2017.19 28287634

[54] Winslow S Lindquist KE Edsjo A Larsson C. The expression pattern of matrix-producing tumor stroma is of prognostic importance in breast cancer. BMC Cancer (2016) 16(1):841. 10.1186/s12885-016-2864-2 27809802 PMC5095990

[55] Liu W Ye H Liu Y Xu C Zhong Y Tian T. Transcriptome-derived stromal and immune scores infer clinical outcomes of patients with cancer. Oncol Lett (2018) 15(4):4351–7. 10.3892/ol.2018.7855 PMC5835954 29541203

[56] Wang H Wu X Chen Y. Stromal-Immune Score-Based Gene Signature: A Prognosis Stratification Tool in Gastric Cancer. Front Oncol (2019) 9:1212. 10.3389/fonc.2019.01212 31781506 PMC6861210

[57] Marvel D Gabrilovich DI. Myeloid-derived suppressor cells in the tumor microenvironment: expect the unexpected. J Clin Invest (2015) 125(9):3356–64. 10.1172/JCI80005 PMC4588239 26168215

[58] Amit A Vijayamahantesh Dikhit MR Singh AK Kumar V Suman SS. Immunization with Leishmania donovani protein disulfide isomerase DNA construct induces Th1 and Th17 dependent immune response and protection against experimental visceral leishmaniasis in Balb/c mice. Mol Immunol (2017) 82:104–13. 10.1016/j.molimm.2016.12.022 28064069

[59] Amit A Dikhit MR Mahantesh V Chaudhary R Singh AK Singh A. Immunomodulation mediated throughLeishmania donovaniprotein disulfide isomerase by eliciting CD8+ T-cell in cured visceral leishmaniasis subjects and identification of its possible HLA class-1 restricted T-cell epitopes. J Biomol Struct Dyn (2016) 35(1):128–40. 10.1080/07391102.2015.1134349 26727289

[60] Caorsi C Niccolai E Capello M Vallone R Chattaragada MS Alushi B. Protein disulfide isomerase A3-specific Th1 effector cells infiltrate colon cancer tissue of patients with circulating anti-protein disulfide isomerase A3 autoantibodies. Transl Res (2016) 171:17–28.e1-2. 10.1016/j.trsl.2015.12.013 26772958

[61] Xiao Y Li C Gu M Wang H Chen W Luo G. Protein Disulfide Isomerase Silence Inhibits Inflammatory Functions of Macrophages by Suppressing Reactive Oxygen Species and NF-κB Pathway. Inflammation (2018) 41(2):614–25. 10.1007/s10753-017-0717-z 29294242

[62] Vinaik R Kozlov G Gehring K. Structure of the non-catalytic domain of the protein disulfide isomerase-related protein (PDIR) reveals function in protein binding. PloS One (2013) 8(4):e62021. 10.1371/journal.pone.0062021 23614004 PMC3629029

[63] Woroniecka KI Rhodin KE Chongsathidkiet P Keith KA Fecci PE. T-cell Dysfunction in Glioblastoma: Applying a New Framework. Clin Cancer Res (2018) 24(16):3792–802. 10.1158/1078-0432.CCR-18-0047 PMC6095741 29593027

[64] Wang D Wang X Si M Yang J Sun S Wu H. Exosome-encapsulated miRNAs contribute to CXCL12/CXCR4-induced liver metastasis of colorectal cancer by enhancing M2 polarization of macrophages. Cancer Lett (2020) 474:36–52. 10.1016/j.canlet.2020.01.005 31931030

[65] Yang D Liu K Fan L Liang W Xu T Jiang W. LncRNA RP11-361F15.2 promotes osteosarcoma tumorigenesis by inhibiting M2-Like polarization of tumor-associated macrophages of CPEB4. Cancer Lett (2020) 473:33–49. 10.1016/j.canlet.2019.12.041 31904478

[66] Sa JK Chang N Lee HW Cho HJ Ceccarelli M Cerulo L. Transcriptional regulatory networks of tumor-associated macrophages that drive malignancy in mesenchymal glioblastoma. Genome Biol (2020) 21(1):216. 10.1186/s13059-020-02140-x 32847614 PMC7448990

[67] Netea-Maier RT Smit JWA Netea MG. Metabolic changes in tumor cells and tumor-associated macrophages: A mutual relationship. Cancer Lett (2018) 413:102–9. 10.1016/j.canlet.2017.10.037 29111350

[68] Zhou W Ke SQ Huang Z Flavahan W Fang X Paul J. Periostin secreted by glioblastoma stem cells recruits M2 tumour-associated macrophages and promotes malignant growth. Nat Cell Biol (2015) 17(2):170–82. 10.1038/ncb3090 PMC4312504 25580734

[69] Goswami S Walle T Cornish AE Basu S Anandhan S Fernandez I. Immune profiling of human tumors identifies CD73 as a combinatorial target in glioblastoma. Nat Med (2020) 26(1):39–46. 10.1038/s41591-019-0694-x 31873309 PMC7182038

[70] Cheng Q Li J Fan F Cao H Dai ZY Wang ZY. Identification and Analysis of Glioblastoma Biomarkers Based on Single Cell Sequencing. Front Bioeng Biotechnol (2020) 8:167. 10.3389/fbioe.2020.00167 32195242 PMC7066068

[71] Litak J Mazurek M Grochowski C Kamieniak P Roliński J. PD-L1/PD-1 Axis in Glioblastoma Multiforme. Int J Mol Sci (2019) 20(21):5347. 10.3390/ijms20215347 PMC6862444 31661771

[72] Liu S Wang Z Wang Y Fan X Zhang C Ma W. PD-1 related transcriptome profile and clinical outcome in diffuse gliomas. Oncoimmunology (2018) 7(2):e1382792. 10.1080/2162402X.2017.1382792 29308304 PMC5749656

[73] Li X Wang B Gu L Gao L Ma C Liang X. Tim-3 expression predicts the abnormal innate immune status and poor prognosis of glioma patients. Clin Chim Acta (2018) 476:178–84. 10.1016/j.cca.2017.11.022 29174343

[74] Zhang J Wang J Marzese DM Wang X Yang Z Li C. B7H3 regulates differentiation and serves as a potential biomarker and theranostic target for human glioblastoma. Lab Invest (2019) 99(8):1117–29. 10.1038/s41374-019-0238-5 30914782

[75] Wang Z Li G Wang Q Bao Z Wang Z Zhang C. PD-L2 expression is correlated with the molecular and clinical features of glioma, and acts as an unfavorable prognostic factor. OncoImmunology (2018) 8(2):e1541535. 10.1080/2162402x.2018.1541535 30713802 PMC6343813

[76] Chen G Huang AC Zhang W Zhang G Wu M Xu W. Exosomal PD-L1 contributes to immunosuppression and is associated with anti-PD-1 response. Nature (2018) 560(7718):382–6. 10.1038/s41586-018-0392-8 PMC6095740 30089911

[77] Cortes J Cescon DW Rugo HS Nowecki Z Im S-A Yusof MM. Pembrolizumab plus chemotherapy versus placebo plus chemotherapy for previously untreated locally recurrent inoperable or metastatic triple-negative breast cancer (KEYNOTE-355): a randomised, placebo-controlled, double-blind, phase 3 clinical trial. Lancet (2020) 396(10265):1817–28. 10.1016/s0140-6736(20)32531-9 33278935

