# Supplementary material for: PDIA5 is Correlated With Immune Infiltration and Predicts Poor Prognosis in Gliomas
Source: Front Immunol. 2021 Feb 16;12:628966. doi: 10.3389/fimmu.2021.628966 (PMC7921737; doi:10.3389/fimmu.2021.628966)
Supplement: Supplementary file 1 [file DataSheet_1.pdf]

Supplementary Material

Supplementary Figures and Tables

Supplementary Figures

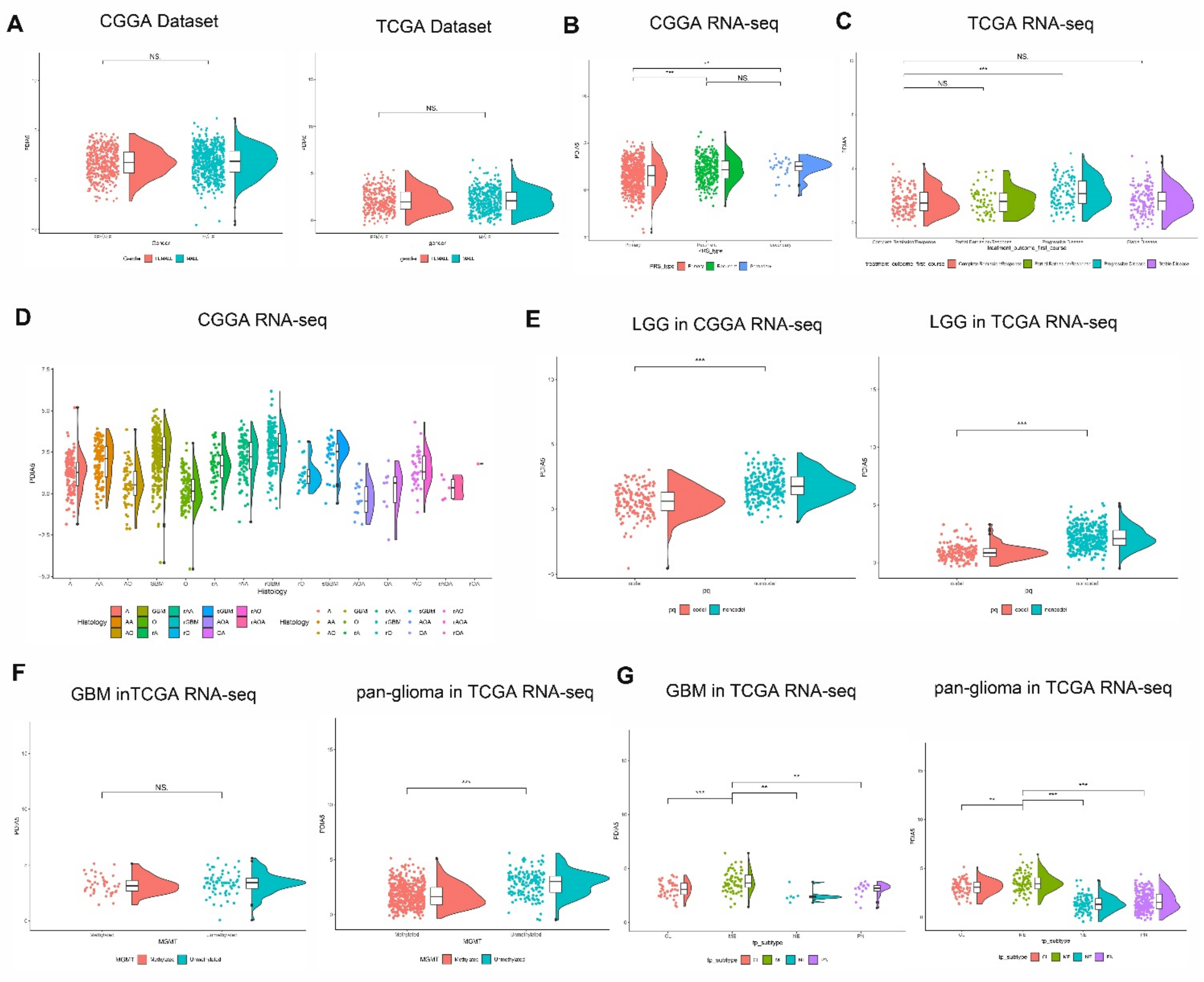

Supplementary Figure S1. Clinical and molecular characteristics of PDIA5 in gliomas. **A**. The relationship between PDIA5 expression and gender in the CGGA and TCGA datasets. **B**. The expression levels of PDIA5 among primary gliomas, recurrent gliomas, and secondary gliomas from the CGGA dataset. **C**. PDIA5 expression in different treatment outcome groups from the TCGA dataset. **D**. The expression levels of PDIA5 increased by the histopathologic classification in the CGGA dataset. **E**. PDIA5 expression was upregulated with 1p/19q non-codeletion status in LGG from the CGGA and TCGA datasets. **F**. PDIA5 expression was upregulated in MGMT promoter unmethylated samples of pan-gliomas but not GBM in the TCGA dataset. **G**. The PDIA5 expression pattern in the molecular

subtypes of GBM and pan-gliomas from TCGA. CL, classical; MES, mesenchymal; PN, pro-neural; NE, neural. ** P <.01, *** P <.001, ns. p>.05.

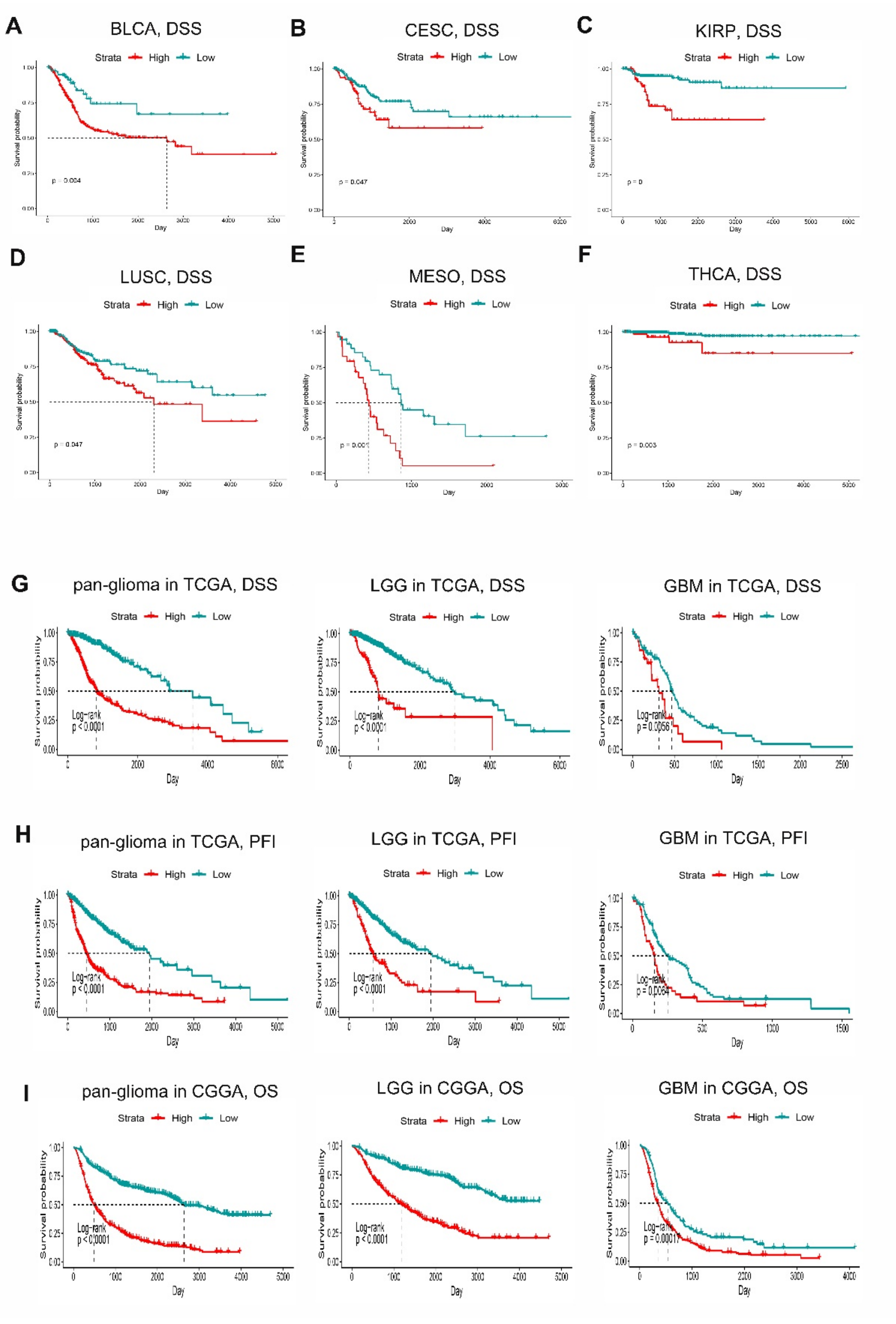

Supplementary Figure S2. Kaplan-Meier survival curves comparing high and low expression of PDIA5 in different cancers using Kaplan-Meier Plotter. DSS of Bladder urothelial carcinoma (BLCA)

(**A**), Cervical and endocervical cancers (CESC) (**B**), Kidney renal papillary cell carcinoma (KIRP) (**C**), Lung squamous cell carcinoma (LUSC) (**D**), Mesothelioma (MESO) (**E**) and Thyroid carcinoma (THCA) (**F**). **G**. Kaplan-Meier analysis of DSS based on high vs low expression of PDIA5 in pan-glioma, LGG and GBM patients in the TCGA dataset. **H**. Kaplan-Meier analysis of PFI based on high vs low expression of PDIA5 in pan-glioma, LGG and GBM patients in the TCGA dataset. **I**. Kaplan-Meier analysis of overall survival (OS) based on high vs low expression of PDIA5 in pan-glioma, LGG and GBM patients in the CGGA dataset. The red curve represents patients with high expression of PDIA5, and the green curve represents patients with low expression of PDIA5. OS, overall survival; DSS, disease specific survival; PFI, progression-free interval.

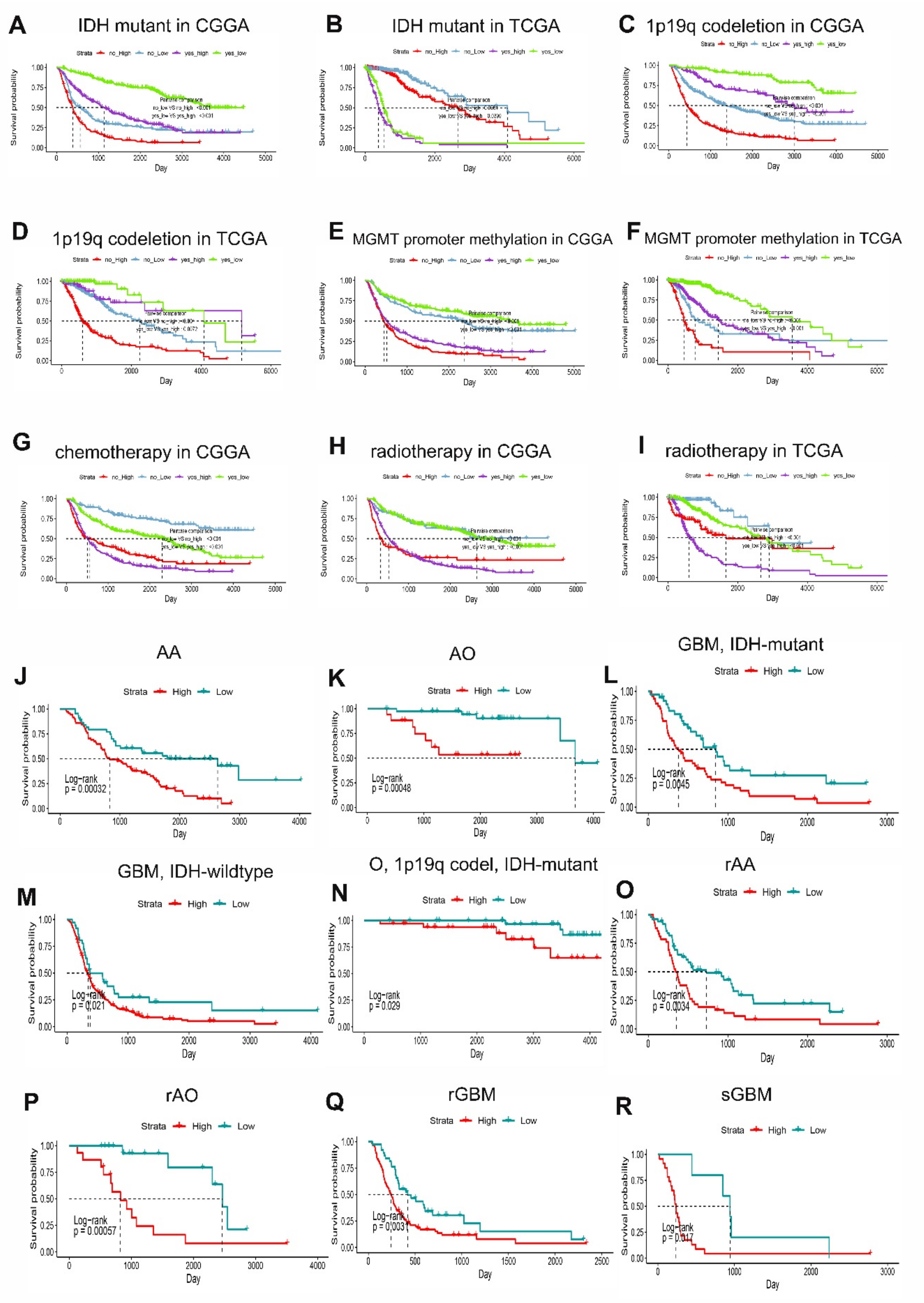

Supplementary Figure S3. Kaplan-Meier survival curves comparing high and low expression of PDIA5 in gliomas based on different molecular markers and treatments. IDH mutant status in CGGA(**A**)

and TCGA(**B**) datasets. 1p19q codeletion status in CGGA(**C**) and TCGA(**D**) datasets. MGMT prompter methylation status in CGGA(**E**) and TCGA(**F**) datasets. Chemotherapy in the CGGA dataset(**G**). Radiotherapy in the CGGA(**H**) and TCGA(**I**) datasets. Kaplan-Meier overall survival curves for patients with anaplastic astrocytoma (AA)(**J**), anaplastic oligodendrocytoma (AO)(**K**), GBM with IDH-mutation(**L**), GBM with wildtype IDH(**M**), oligodendroglioma(O) with 1p/19q co-deletion and IDH-mutation(**N**), recurrent anaplastic astrocytoma (rAA)(**O**), recurrent anaplastic oligodendrocytoma (rAO)(**P**), recurrent GBM (rGBM)(**Q**) and secondary GBM (sGBM)(**R**). The red curve represents patients with high expression of PDIA5, and the green curve represents patients with low expression of PDIA5.

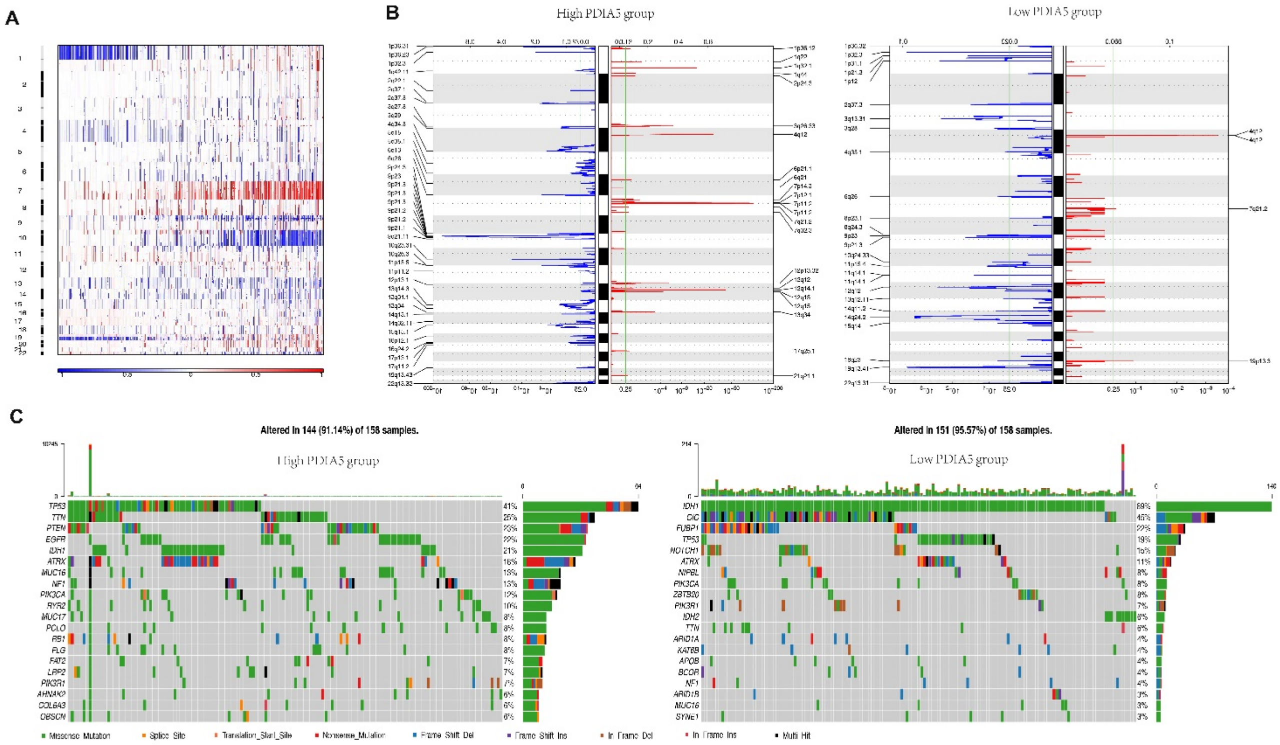

Supplementary Figure S4. PDIA5 expression correlates with distinct genomic alterations. **A**. Overall copy number variation (CNV) profile according to high vs low PDIA5 expression. **B**. GISTIC 2.0 amplifications and deletions in gliomas with high and low PDIA5 expression. **C**. Differential somatic mutations were detected in gliomas with high and low PDIA5 expression. Red represents amplification and blue represents deletion.

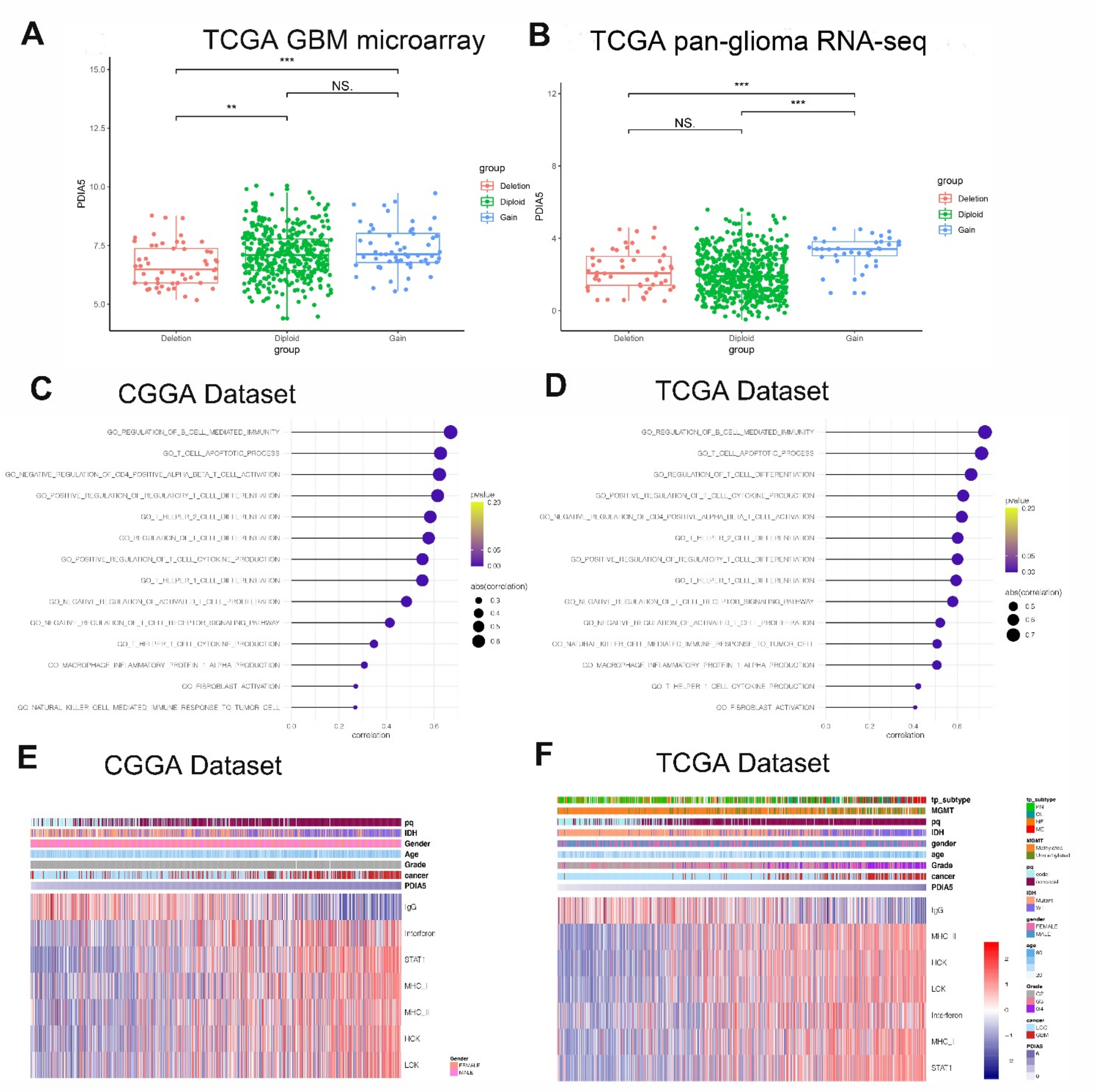

Supplementary Figure S5. Relationship between PDIA5 expression and PDIA5 copy number in **A**. GBM microarray data from the TCGA dataset. **B.** pan-glioma RNA-seq data from the TCGA dataset. * P <.05, ** P <.01, *** P <.001, ns. p>.05. **C, D**. Correlation of PDIA5 and immunity pathways in the CGGA and TCGA datasets. **E, F**. The relationship between PDIA5 and inflammatory activities in the CGGA and TCGA datasets. Expression values are z-transformed and are highlighted in red for high expression and blue for low expression as indicated in the scale bar.

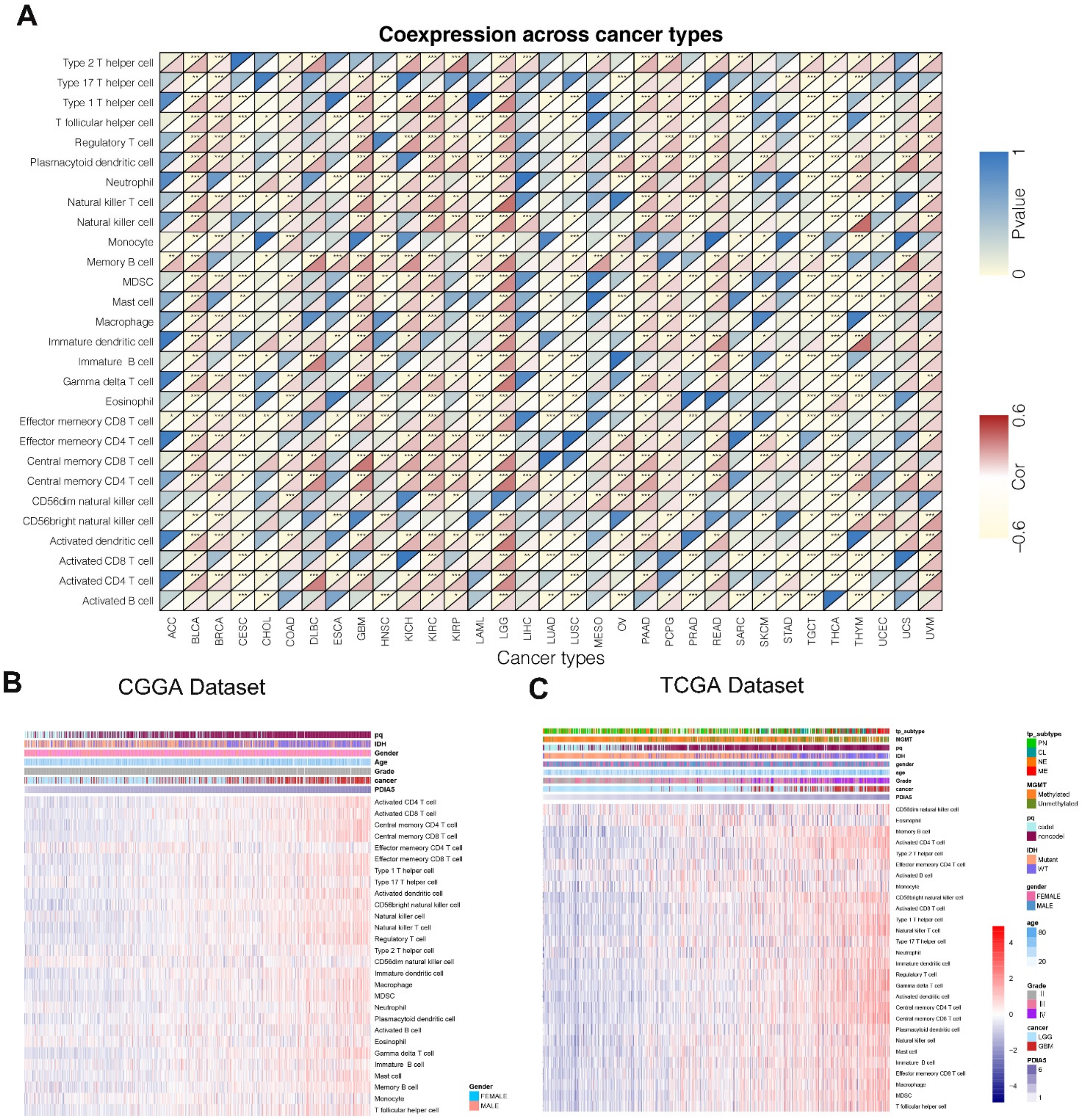

Supplementary Figure S6. Correlation between PDIA5 expression and immune cell infiltration in gliomas. **A**. Relationship between PDIA5 expression and immune infiltration in 33 cancer types. **B**. Correlation of PDIA5 and 28-immune cell lineage genes in pan-glioma analysis in the CGGA dataset. **C**. Correlation of PDIA5 and 28-immune cell lineages genes in pan-glioma analysis in the the TCGA dataset. Expression values are z-transformed and are highlighted in red for high expression and blue for low expression as indicated in the scale bar.

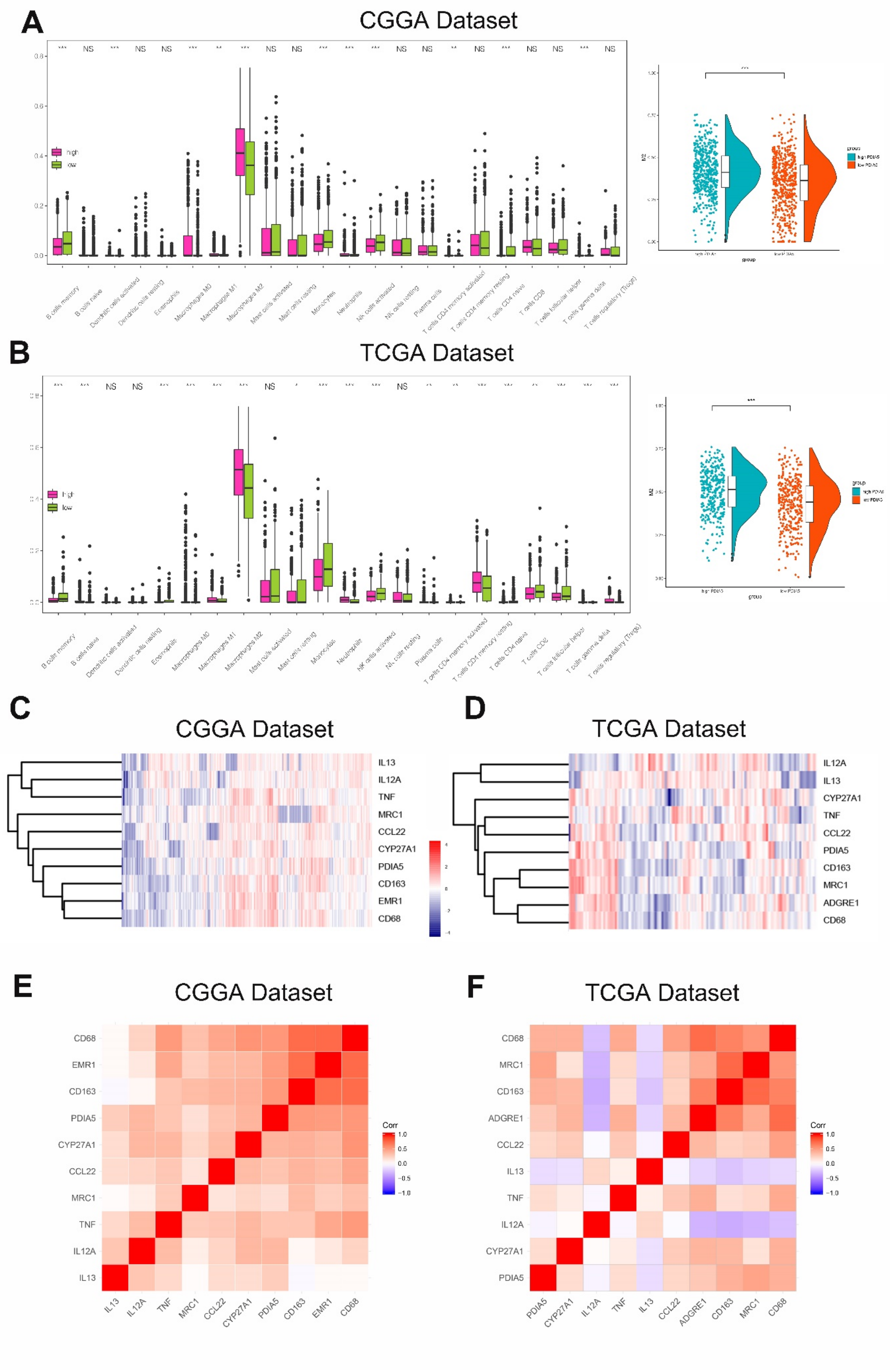
A
CGGA Dataset
high
low
B cells memory
B cells naive
Dendritic cells activated
Dendritic cells resting
Eosinophils
Macrophages M0
Macrophages M1
Macrophages M2
Mast cells activated
Mast cells resting
Monocytes
Neutrophils
NK cells activated
NK cells resting
Plasma cells
T cells CD4 memory activated
T cells CD4 memory resting
T cells CD4 naive
T cells CD8
T cells follicular helper
T cells gamma delta
T cells regulatory (Tregs)
B
TCGA Dataset
C
CGGA Dataset
IL13
IL12A
TNF
MRC1
CCL22
CYP27A1
PDIA5
CD163
EMR1
CD68
D
TCGA Dataset
IL12A
IL13
CYP27A1
TNF
CCL22
PDIA5
CD163
MRC1
ADGRE1
CD68
E
CGGA Dataset
Corr
1.0
0.5
0.0
−0.5
−1.0
F
TCGA Dataset

Supplementary Figure S7. The association between PDIA5 and macrophages in gliomas. The differences in expression value for 22 immune cells between high and low expression of PDIA5 group in the CGGA (**A**) and TCGA (**B**) datasets. Heatmap of PDIA5 and macrophage markers in gliomas from the CGGA (**C**) and TCGA (**D**) datasets. Correlation analysis between PDIA5 and macrophage markers in the CGGA (**E**) and TCGA (**F**) datasets.

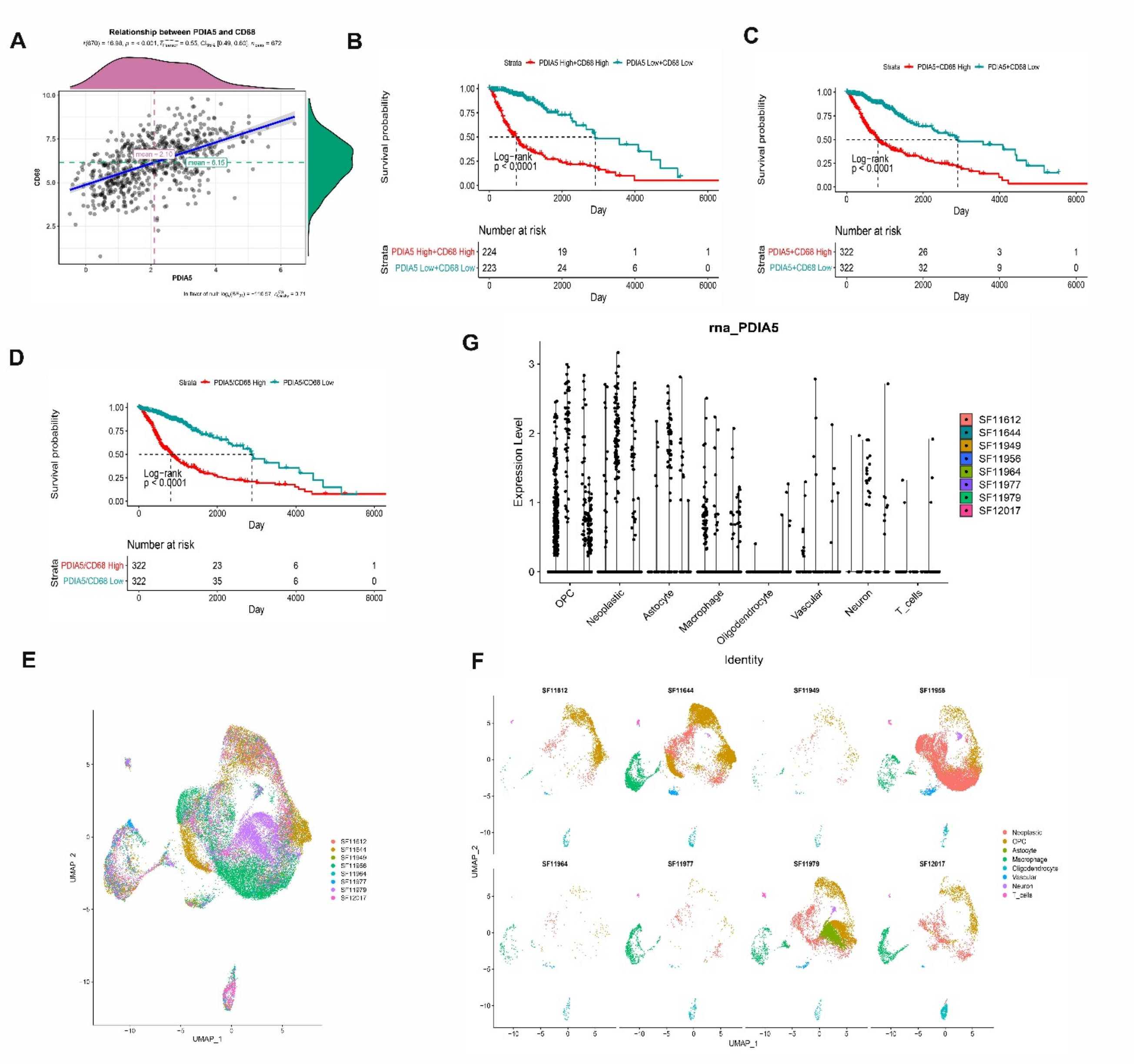

Supplementary Figure S8. The relationship between PDIA5 and CD68 as well as the scRNA-seq results of PDIA5 in gliomas. **A.** correlation analysis between PDIA5 and CD68 in gliomas. **B**. Kaplan-Meier analysis of OS based on high vs low expression of PDIA5 and CD68 in gliomas. **C**. Kaplan-Meier analysis of OS based on high vs low combined sum of PDIA5 and CD68 expression in gliomas. **D**. Kaplan-Meier analysis of OS based on high vs low ratio of PDIA5 to CD68 in gliomas. The red curve represents high expression, and the blue curve represents low expression. **E**. Representative merged image showing the data from 8 glioma samples. **F**. Eight clusters of cells, including neoplastic cells, oligodendrocyte precursor cells (OPC), astrocytes, macrophages, oligodendrocytes, vascular

endothelial cells, neurons, and T cells were identified from the eight glioma samples. **G**. Violin plot of the PDIA5 expression distribution of different glioma samples.

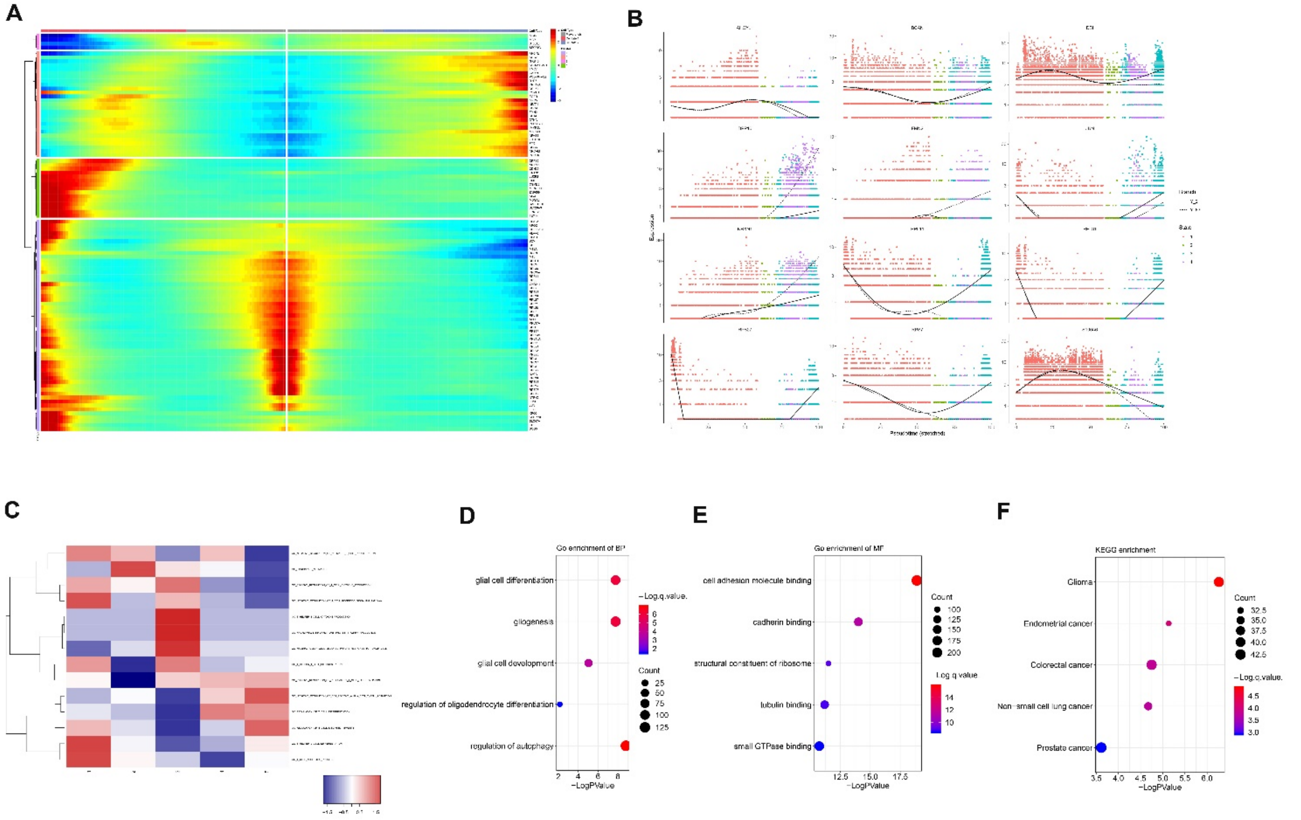

Supplementary Figure S9. Single-cell pseudotime trajectories and functional annotations of neoplastic cells in gliomas. **A**. 100 genes with branch-dependent expression for branch point 1 were identified. **B**. the top 12 genes with branch-dependent expression for branch point 1. **C**. GSEA of neoplastic cells for PDIA5 in each state. The red color represents positive correlation, and the blue color represents negative correlation. **D.** Representative images of biological processes (BP) from GO enrichment analysis based on PDIA5 in neoplastic cells. **E**. Representative image of molecular functions (MF) from GO enrichment analysis based on PDIA5 in neoplastic cells. **F**. Representative image of KEGG pathway analysis based on PDIA5 in neoplastic cells.

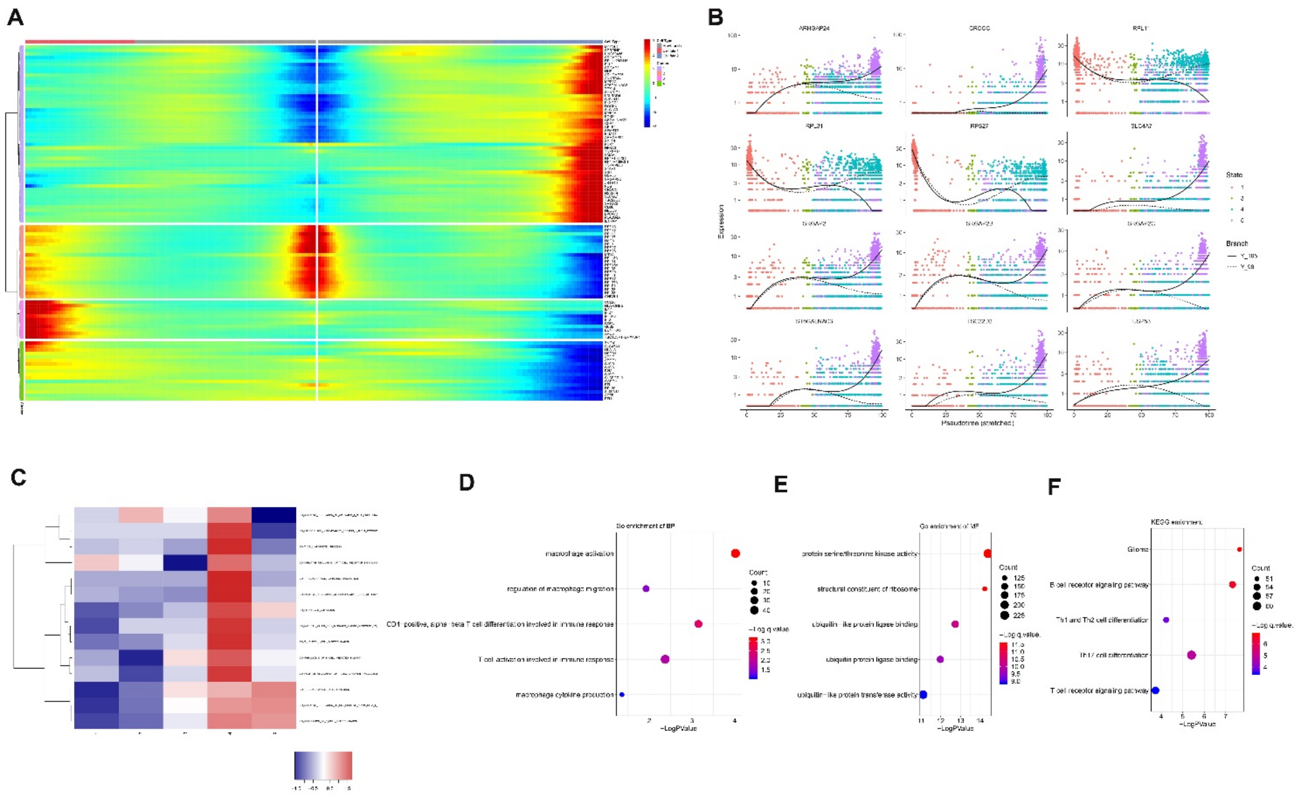

Supplementary Figure S10. The Single-cell pseudotime trajectories and functional annotations of macrophages in gliomas. **A**. 100 genes with branch-dependent expression for branch point 2 were identified. **B**. the top 12 genes with branch-dependent expression for branch point 2. **C**. GSEA with macrophages for PDIA5 in each state. The red color represents positive correlation, and the blue color represents negative correlation. **D.** Representative image of biological processes (BP) from GO enrichment analysis based on PDIA5 in macrophage. **E**. Representative image of molecular functions (MF) from GO enrichment analysis based on PDIA5 in macrophages. **F**. Representative image of KEGG pathway analysis based PDIA5 in macrophages.

Supplementary Tables

Table S1. Demographics and clinical characteristics of gliomas patients from Xiangya Hospital

Table S2. The correlation between PDIA5 and GO pathways in gliomas.

Table S3. GO enrichment analysis of biological process based on PDIA5 in neoplastic cells.

Table S4. GO enrichment analysis of molecular function based on PDIA5 in neoplastic cells

Table S5. KEGG pathway analysis based on PDIA5 in neoplastic cells.

Table S6. GO enrichment analysis of biological process based on PDIA5 in macrophages.

Table S7. GO enrichment analysis of molecular function based on PDIA5 in macrophages.

Table S8. KEGG pathway analysis based on PDIA5 in macrophages.

Table S1 Demographics and clinical characteristics of gliomas patients from Xiangya Hospital

Wax code	Pathological Level in slices	XYNS Tissue bank code	Gender	Age	Lesion Location	Score	PDIA5 Expression Area	PDIA5 Expression Intensity
GT1	I	130805W35G1	M	34	R-front lobe	0	0	0
GTR2	IV	130816W37G1R	M	44	L-parieto lobe	9	3	3
GT3	II	130819W35G1	M	65	L-parieto lobe	3	1	3
GT4	II	130821W37G1	M	43	R-front lobe	3	1	3
GT5	II	130822W37G2	M	29	cauda cerebelli	1	1	1
GT6	IV	130829W37G1	M	74	R-frontal-parieto lobe	9	3	3
GT7	IV	130830W35G2	M	44	L-frontal lobe	6	2	3
GT8	IV	130918W37G3	M	75	L-front lobe	6	2	3
GT10	II	130926W37G1	M	50	R-front lobe	4	2	2
GT13	III	130929W35G3	M	64	R-temporal-insular lobe	0	2	1
GTR14	II	130929W37G1R	M	43	R-frontal lobe	4	2	2
GT15	III	130930W35G1	M	71	R-parieto-occipital lobe	4	2	2
GT17	IV	131014W37G1	F	69	L-occipital lobe	6	2	3
GT18	II	131015W37G1	M	17	L-temporal lobe	0	2	0
GT20	III	131029W37G1	M	55	R-temporal lobe	2	1	2
GT22	III	131031W37G2	M	54	L-frontal lobe	4	2	2
GT23	III	131104W39G1	M	42	L-frontal lobe	4	2	2
GT24	III	131108W37G1	M	43	L-frontal lobe	2	2	1
GT28	III	131114W39G2	M	36	R-frontal lobe	6	2	3
GT29	IV	131115W35G1	M	34	R-temporal lobe	9	3	3
GT30	II	131115W36G1	M	1.4	R-temporal lobe	2	1	2
GT31	I	131115W36G2	M	42	L-front lobe	0	0	2
GT32	II	131115W37G1	F	45	L-front lobe	0	2	0
GTR34	III	131118W37G1R	F	36	R-frontal lobe	4	2	2
GT35	IV	131120W37G1	M	61	L-parieto lobe	9	3	3
GT38	IV	131121W37G1	M	72	L-temporal-parieto lobe	6	2	3
GT40	III	131125W37G2	F	37	R-parieto lobe	6	2	3
GT42	IV	131202W37G1	F	74	R-parieto-occipital lobe	9	3	3
GT43	III	131204W37G1	M	39	L-frontal lobe	4	2	2
GT45	IV	131206W39G1	M	39	R-frontal lobe	4	2	2
GT47	II	131211W37G1	M	34	L-frontal-temporal lobe	4	2	2

Table S2 The correlation between PDIA5 and GO pathways in gliomas

	symbol	correlation in pan-glioma	pvalue in pan-glioma	correlation in GBM	pvalue in GBM
7349	PDIA5	1	0	1	0
5235	GO_REGULATION_OF_B_CELL_MEDIATED_IMMUNITY	0.72731205	1.24095366106935E-111	0.541056131	8.80786059274451E-13
3343	GO_NEGATIVE_REGULATION_OF_T_CELL_RECEPTOR_SIGNALING_PATHWA	0.57993449	1.20057760266358E-61	0.405101749	2.71623075536244E-07
4607	GO_POSITIVE_REGULATION_OF_REGULATORY_T_CELL_DIFFERENTIATIO	0.6018193	1.90784483807895E-67	0.333375675	3.06293434741598E-05
2767	GO_NEGATIVE_REGULATION_OF_CD4_POSITIVE_ALPHA_BETA_T_CELL_ACTIVATION	0.62129118	5.37253782850929E-73	0.569814953	2.74026136051458E-14
6227	GO_REGULATION_OF_T_CELL_DIFFERENTIATION_IN_THYM	0.26718887	1.89704903537395E-12	0.480029141	5.10581401238836E-10
7031	GO_T_HELPER_2_CELL_DIFFERENTIATION	0.60256428	1.18905011290499E-67	0.452043145	6.36768065510894E-09
7026	GO_T_HELPER_1_CELL_DIFFERENTIATION	0.59534351	1.1039410094545E-65	0.500669951	6.82419645461363E-11
4694	GO_POSITIVE_REGULATION_OF_T_CELL_CYTOKINE_PRODUCTION	0.62760136	7.02053185576331E-75	0.481209816	4.566929614055E-10
7009	GO_T_CELL_APOPTOTIC_PROCESS	0.71148028	9.42603182420773E-105	0.48467418	3.28412848258736E-10
7025	GO_T_HELPER_1_CELL_CYTOKINE_PRODUCTION	0.42201263	2.11731315029073E-30	0.230060933	0.00462317
2217	GO_MACROPHAGE_INFLAMMATORY_PROTEIN_1_ALPHA_PRODUCTION	0.5075601	2.76532058961897E-45	0.292235481	0.000284807
2668	GO_NATURAL_KILLER_CELL_MEDIATED_IMMUNE_RESPONSE_TO_TUMOR	0.50862158	1.69553036568869E-45	0.272100885	0.000755919
1506	GO_FIBROBLAST_ACTIVATION	0.40851581	2.04405857801024E-28	0.360676245	5.788498640275E-06
2688	GO_NEGATIVE_REGULATION_OF_ACTIVATED_T_CELL_PROLIFERATION	0.52257206	2.32592217318822E-48	0.330896386	3.53635069235981E-05

Table S3 GO enrichment analysis of biological process based on PDIA5 in neoplastic cells

ID	Description	GeneRatio	BgRatio	pvalue	p.adjust	qvalue
GO:0010001	glial cell differentiation	91/4591	218/18670	1.68E-08	1.31E-06	9.84E-07
GO:0042063	gliogenesis	114/4591	290/18670	1.70E-08	1.31E-06	9.84E-07
GO:0021782	glial cell development	50/4591	116/18670	9.16E-06	0.0002426	0.0001829
GO:0048713	regulation of oligodendrocyte differentiation	17/4591	39/18670	0.007194	0.0441991	0.0333133
GO:1904925	positive regulation of autophagy of mitochondrion in response to mitochondrial depolarization	6/4591	13/18670	0.074763	0.224849	0.1694709

Table S4 GO enrichment analysis of molecular function based on PDIA5 in neoplastic cells

ID	Description	GeneRatio	BgRatio	pvalue	p.adjust	qvalue
GO:0050839	cell adhesion molecule binding	221/4577	499/17697	1.30E-19	1.52E-16	1.27E-16
GO:0045296	cadherin binding	150/4577	331/17697	9.84E-15	5.75E-12	4.80E-12
GO:0003735	structural constituent of ribosome	98/4577	202/17697	3.20E-12	1.25E-09	1.04E-09
GO:0015631	tubulin binding	144/4577	336/17697	6.60E-12	1.93E-09	1.61E-09
GO:0031267	small GTPase binding	178/4577	443/17697	1.86E-11	4.36E-09	3.64E-09

Table S5 KEGG pathway analysis based on PDIA5 in neoplastic cells

ID	Description	GeneRatio	BgRatio	pvalue	p.adjust	qvalue
hsa05214	Glioma	41/2206	75/8041	5.41E-07	2.21E-05	1.43E-05
hsa05213	Endometrial cancer	32/2206	58/8041	7.31E-06	0.000138499	8.92E-05
hsa05210	Colorectal cancer	42/2206	86/8041	1.79E-05	0.000261462	0.000168382
hsa05223	Non-small cell lung cancer	35/2206	68/8041	2.13E-05	0.000298343	0.000192133
hsa05215	Prostate cancer	43/2206	97/8041	0.000244016	0.00187079	0.001204791

Table S6 GO enrichment analysis of biological process based on PDIA5 in macrophages

ID	Description	GeneRatio	BgRatio	pvalue	p.adjust	qvalue
GO:0042116	macrophage activation	49/6086	95/18670	9.62E-05	0.001011106	0.000683085
GO:1905521	regulation of macrophage migration	20/6086	39/18670	0.011876182	0.04770489	0.032228552
GO:0002294	CD4-positive, alpha-beta T cell differentiation involved in immune response	32/6086	60/18670	0.000708894	0.005057352	0.003416655
GO:0002287	alpha-beta T cell activation involved in immune response	32/6086	61/18670	0.001029014	0.0069048	0.004664757
GO:0010934	macrophage cytokine production	9/6086	16/18670	0.043466958	0.125205854	0.084586787

Table S7 GO enrichment analysis of molecular function based on PDIA5 in macrophages

ID	Description	GeneRatio	BgRatio	pvalue	p.adjust	qvalue
GO:0004674	protein serine/threonine kinase activity	231/6128	439/17697	4.05E-15	3.53E-12	2.66E-12
GO:0003735	structural constituent of ribosome	124/6128	202/17697	5.95E-15	3.53E-12	2.66E-12
GO:0044389	ubiquitin-like protein ligase binding	169/6128	308/17697	1.87E-13	7.39E-11	5.57E-11
GO:0031625	ubiquitin protein ligase binding	159/6128	290/17697	1.05E-12	3.12E-10	2.35E-10
GO:0019787	ubiquitin-like protein transferase activity	207/6128	407/17697	7.76E-12	1.84E-09	1.39E-09

Table S8 KEGG pathway analysis based on PDIA5 in macrophages

ID	Description	GeneRatio	BgRatio	pvalue	p.adjust	qvalue
hsa05214	Glioma	51/2923	75/8041	2.25E-08	2.70E-07	1.41E-07
hsa04662	B cell receptor signaling pathway	54/2923	82/8041	4.80E-08	5.34E-07	2.79E-07
hsa04658	Th1 and Th2 cell differentiation	52/2923	92/8041	5.89E-05	0.000267764	0.00013962
hsa04659	Th17 cell differentiation	62/2923	107/8041	3.92E-06	2.69E-05	1.40E-05
hsa04660	T cell receptor signaling pathway	56/2923	104/8041	0.000186709	0.000701244	0.000365648
